# Sliding physical invariant neural operator for long-term prediction of complex dynamics in physical systems

**DOI:** 10.1093/nsr/nwag027

**Published:** 2026-01-27

**Authors:** Yanjie Wang, Ying Li, Yaxin Peng, Shihui Ying

**Affiliations:** Department of Mechanics, School of Mechanics and Engineering Science, Shanghai University, Shanghai 200444, China; Department of Computer Science and Technology, Computer Engineering and Science, Shanghai University, Shanghai 200444, China; Department of Mathematics, School of Science, Shanghai University, Shanghai 200444, China; Department of Mechanics, School of Mechanics and Engineering Science, Shanghai University, Shanghai 200444, China; Shanghai Institute of Applied Mathematics and Mechanics, Shanghai University, Shanghai 200444, China

**Keywords:** neural operator, partial differential equation, sliding physical invariant, feature mixture module, feature interaction module

## Abstract

Operator learning aims to simulate the underlying physical system to solve an entire family of partial differential equations (PDEs), rather than focusing on a single instance of an equation. Current approaches encode initial conditions into physical invariants to guide the solution of multiple equations; however, these static physical invariants capture only short-term dynamics and fail to represent long-term evolution, thereby degrading both accuracy and stability over time. In this paper, we introduce the sliding physical invariant neural operator, which integrates physical invariants that slide dynamically with the evolution of the underlying PDEs. Compared with existing methods, our approach achieves substantial error reductions of 34.3%–79.8% in the training domain and 7.7%–76.5% in the future domain, underscoring its markedly enhanced generalization and long-term predictive performance across a wide range of PDE scenarios.

## Teaser

We propose the Sliding Physical Invariant Neural Operator (SPINO), which integrates sliding physical invariants to improve long-term accuracy, stability, and generalization in operator learning.

## INTRODUCTION

Partial differential equations (PDEs) serve as the mathematical language for describing the intricate phenomena observed in the physical world, with widespread applications across various domains, ranging from neuronal excitations to turbulent flows and even global climate dynamics [1,2]. Over the past decade, traditional PDE solvers, such as the finite difference method (FDM) [3] and the finite element method (FEM) [4], have been widely used in practice. These methods play a crucial role in computational fluid dynamics (CFD) applications [5], providing valuable insights into the behavior of complex systems governed by PDEs. However, inherent limitations associated with these techniques—such as restrictions on step size [6] and the curse of dimensionality [7,8] when solving high-dimensional PDEs—have impeded further advancements. In addition, these methods are often time-consuming and computationally intensive, as they require recomputation for each new equation, making them less practical for real-world applications. Consequently, an increasing number of studies [9–11] have focused on developing efficient methods for solving families of PDEs by exploring effective and computationally efficient representations of the underlying physical systems.

Benefiting from recent advances in optimization, high-performance computing and GPU-based hardware, deep learning has demonstrated strong performance in information processing and prediction, with widespread applications in image analysis [12], natural language processing [13] and recommendation systems [14]. In the emerging field of scientific machine learning (SciML), deep learning has become a powerful tool for operator learning, effectively capturing complex mappings between function spaces in PDE problems. Compared with traditional PDE solvers, these data-driven approaches can achieve orders-of-magnitude speed-ups by bypassing iterative solution procedures and directly modeling equation dynamics from data. The pioneering work DeepONet [15] introduces the first practical realization of the universal operator approximation theorem [16] and has been employed to tackle challenging problems involving complex, high-dimensional dynamical systems [17–19]. In addition, extensions of DeepONet have recently been proposed in transfer learning [20], multi-fidelity learning [21,22] and reinforcement learning [23].

Another class of neural operators is the integral operators. Specifically, Fourier neural operators (FNOs) [24–26] define a new neural operator by parameterizing the integral kernel directly in Fourier space, providing an expressive and efficient architecture that enhances representational capacity. Although the FNO offers an efficient spectral representation, its reliance on the Fourier transform limits its applicability to periodic and integrable signals. To overcome this limitation, alternatives such as the wavelet neural operator (WNO) [27] and Laplace neural operator (LNO) [28] have been proposed to better capture localized spatial features and transient dynamics. Recently, Transformers [29] have prevailed in neural operator architectures. The Galerkin Transformer [30] employs the Galerkin-type attention mechanism as kernel integral operators. GNOT [31] is a scalable transformer-based neural operator that flexibly handles multiple input functions and irregular meshes via a novel heterogeneous normalized attention mechanism. FactFormer [32] introduces an axial factorized kernel integral that decomposes the input function into multiple sub-functions with one-dimensional domains, effectively reducing computational cost. These advances reflect the growing diversity of neural operator designs and their increasing capacity to model complex systems efficiently.

While neural operators excel in solving parametric PDEs, their reliance on high-quality data and lack of physical consistency limit their reliability in scientific applications. Recent methods, such as PI-DeepONet [33], SepONet [34] and PINO [35], address this by integrating physical constraints into operator learning, thereby improving generalization, efficiency and scalability, particularly in data-limited or complex PDE scenarios. Furthermore, the PDE-preserved neural network (PPNN) [36] incorporates physical prior knowledge by embedding the discretized governing equations directly into the neural network architecture. This approach contrasts sharply with the methods mentioned above, where the physical laws are enforced as soft constraints within the loss functions [33–35].

All of the methods mentioned above share a common goal: to model different PDEs using the same set of model parameters, thereby significantly reducing the time spent on repetitive calculations. However, PDEs are inherently complex, and even minor differences in the equations can lead to significant discrepancies in their solutions. As a result, using the same parameters across diverse PDEs can be challenging, as it becomes difficult to capture the unique characteristics and long-term dynamics of each equation. To address this problem, the physical invariant attention neural operator (PIANO) was introduced by Zhang *et al.* [37]. PIANO employs contrastive learning to extract physical invariants (PIs) from the initial frames of PDE fields, which are then used to generate distinct network parameters for each equation, thereby improving generalization and accuracy over previous neural operator methods. In parallel, recent developments have explored deep learning architectures beyond neural operators for jointly modeling systems with varying parameters. For instance, a ResNet has been employed to recursively predict the evolution of the three-parameter Lorenz system [38] and has also been extended to simultaneously solve Fokker–Planck equations with multiple continuous parameters [39]. These efforts illustrate the potential of leveraging flexible neural architectures to handle more diverse dynamical regimes, thereby complementing neural operator approaches.

Motivated by the evolving nature of time-dependent systems, we pose a natural question: is it possible to learn phase-specific model parameters to improve the modeling of temporal dynamics across different types of PDEs? To that end, we propose the sliding physical invariant neural operator (SPINO), which integrates sliding physical invariants (SPIs) into neural operators via dynamic convolutional (DyConv) kernels [40], enabling stable long-term prediction of system behaviors. Here, unlike the conventional notion of invariants as physical quantities that remain constant over time, SPIs are defined as latent embeddings extracted from physical fields in PDE solutions and are dynamically recomputed at each prediction stage, allowing the model to continuously track the evolving system states.

**Figure 1.**
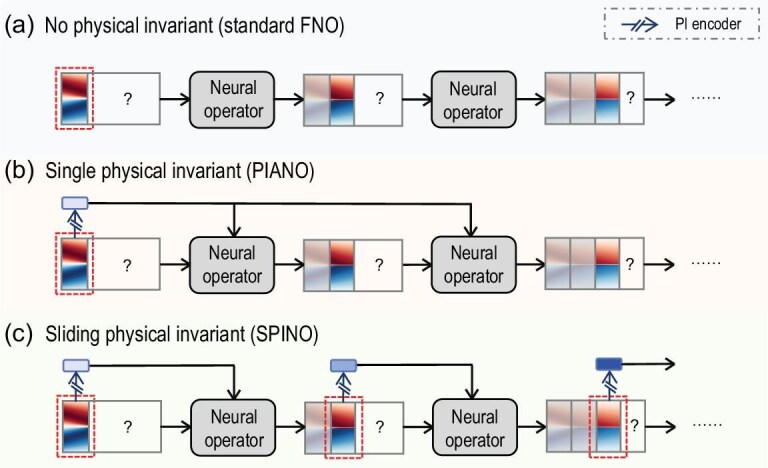
Comparison among standard FNO, PIANO and our SPINO. (a) A standard FNO lacks PIs to guide the operator in solving PDEs. (b) PIANO utilizes static PIs to guide the operator in classifying equations with different physical properties. (c) SPINO, in contrast, employs sliding PIs from various timeframes to guide the operator in capturing the time-varying physical characteristics of the system across different time steps.

To better illustrate the mechanism of our method, we compare three different approaches in Fig. 1. In the case of PIANO [37], it relies solely on static PIs to guide the neural operator in predicting solutions at later times, causing its fixed representation to become progressively misaligned with the dynamically evolving system states. In contrast, our approach utilizes adjacent inputs to generate temporal PIs, which then steer the operator toward capturing the current evolutionary trend of the system. Our main contributions are as follows.

- *Integration of SPIs.* We propose a novel framework that integrates SPIs to learn distinct model parameters for different equations across prediction phases.
- *Feature mixture module (FMM).* We introduce FMM to improve the quality and robustness of SPIs by incorporating a refinement module and a stratified batch-sampling strategy, thereby mitigating error accumulation in sequential prediction.
- *Feature interaction module (FIM).* We introduce FIM in the final block of the SPINO architecture to enhance information exchange between SPIs and high-level features.
- *Comprehensive evaluation on PDE benchmarks.* We validate our approach on five challenging PDE systems—including two one-dimensional (1D) Burgers’ variants, the 1D convection-diffusion equation, the 2D Navier–Stokes equation and the 2D Kolmogorov flow—and demonstrate substantially higher accuracy than baselines, highlighting the robustness of our framework across diverse spatial and physical complexities.

## SPINO FRAMEWORK

In this section, we present the overall framework of SPINO and provide detailed descriptions of its key components.

### Problem formulation

We consider a time-dependent PDE system, which can be formulated as


(1) \begin{eqnarray*}
\left\{ {\begin{array}{l}
\partial _t \mathrm{u}=\mathcal {R}(\mathrm{u}, \theta _{\mathcal {R}}),\\
\mathrm{u}(\mathbf {x}, 0) = \mathrm{u}_0(\mathbf {x}), \ \ (\mathbf {x}, t) \in \Omega \times [0, T],\\
\mathcal {B}[\mathrm{u}](\mathbf {x}, t, \theta _{\mathcal {B}})=0,\end{array}} \right.
\end{eqnarray*}

where $\mathcal {R}$ is the differential operator with parameter $\theta _{\mathcal {R}} \in \Theta _{\mathcal {R}}$, $\mathcal {B}[\mathrm{u}]$ is the boundary condition governed by the parameter $\theta _{\mathcal {B}} \in \Theta _{\mathcal {B}}$ and the product space $\Theta := \Theta _{\mathcal {R}} \times \Theta _{\mathcal {B}}$ denotes the global parameters of the PDE system. The entire PDE system is defined on the domain $\Omega$ over a varying time horizon *T*, and we define $\mathrm{{\bf u}}_{k,t}[\Omega ] := [\mathrm{u}_k[\Omega ], \ldots , \mathrm{u}_{k+t-1}[\Omega ]]$ to denote *t* frames $(t \in \mathbb {N}^{+})$ of PDE fields in $\Omega$. For brevity, we write $\mathrm{{\bf u}}_{k,t}$ in subsequent discussions. By randomly sampling varying $\theta ^{i} \in \Theta$, corresponding to different PDE instances *i* with associated initial conditions $\mathbf {u}_{0}^{i}$, we generate and organize the datasets into $\mathbb {D}_{\mathrm{train}}$, $\mathbb {D}_{\mathrm{val}}$ and $\mathbb {D}_{\mathrm{test}}$. The parameters $\theta ^i$ are used solely for data preparation and are not accessible during training or testing. After training on $\mathbb {D}_{\mathrm{train}}$, the model is evaluated on unseen equations from $\mathbb {D}_{\mathrm{test}}$.

Given the initial *t* frames of PDE fields $\mathrm{{\bf u}}_{0,t}^i$, the task is to predict the remaining fields $\mathrm{{\bf u}}_{t,T - t}^i$ in an auto-regressive manner. Specifically, this prediction process follows a discrete-time dynamical formulation, in which the future states of the system are recursively generated by applying a neural operator $\mathcal {G}$ to the current state. Mathematically, given the ambient space $\mathcal {X}$ and the state space $\mathcal {S} \subset \mathcal {X}$, the dynamical system can be formulated as


(2) \begin{eqnarray*}
{\bf u}_{k+t,t} = \mathcal {G}({\bf u}_{k,t}), \qquad k=0,t,2t,\dots ,
\end{eqnarray*}

where ${\bf u}_{k,t} \in \mathcal {S}$ is the system’s current state, governed by Equation (1). The operator $\mathcal {G}:\mathcal {S} \rightarrow \mathcal {S}$ describes the evolution from ${\bf u}_{k,t}$ to the successive state ${\bf u}_{k+t,t}$.

### Overview

To improve the modeling of time-dependent PDE systems, we introduce the SPINO. SPINO generates phase-specific physical information in the form of SPIs, which enable the model to derive phase-adaptive parameters. This allows it to effectively leverage temporal variations in system dynamics, thereby enhancing the stability of long-term predictions.

**Figure 2.**
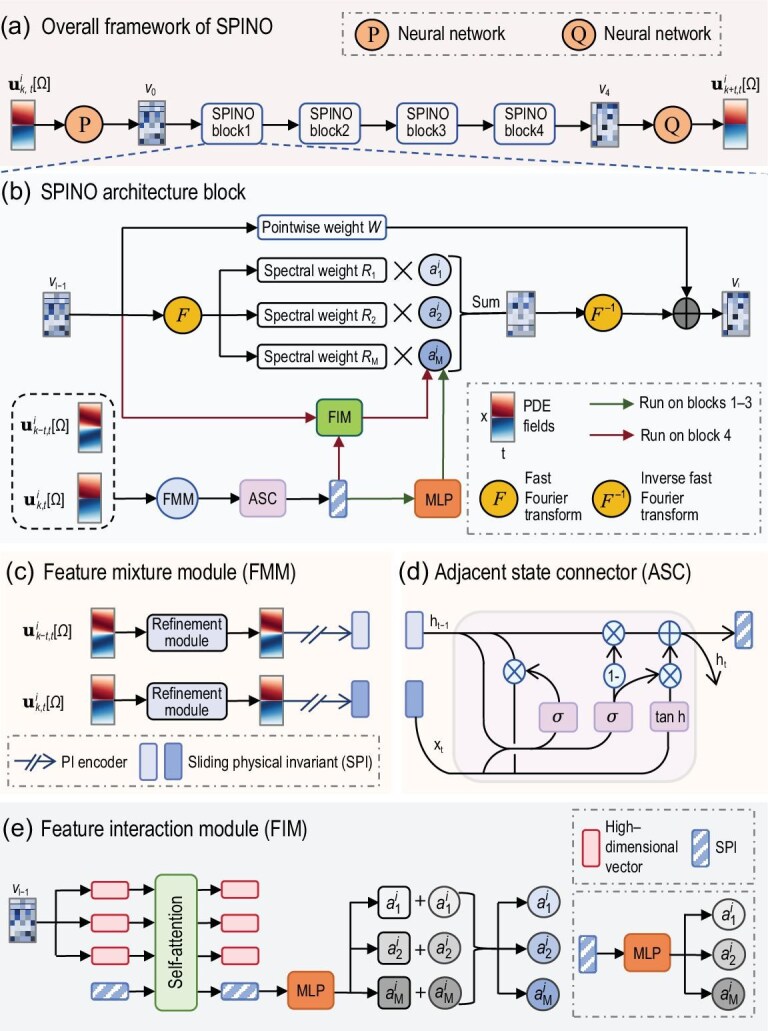
An overview of the SPINO architecture. (a) The overall SPINO pipeline for time-dependent PDE forecasting. Given the *i*th input $\mathbf {u}_{k,t}^i[\Omega ]$, the encoder network *P* generates the initial feature $v_0$, which is progressively refined by four stacked SPINO blocks (1–4) to produce the final feature $v_4$. The decoder network *Q* then maps $v_4$ to the target output $\mathbf {u}_{k+t,t}^i[\Omega ]$. (b) Structure of a single SPINO block. The input feature $\nu _{l-1}$ is split into two branches. In the spectral branch, $\nu _{l-1}$ is transformed to the frequency domain via FFT and processed by spectral weight matrices $(R_1,\ldots ,R_M)$. Their outputs are linearly combined with coefficients $(a_1^i,\ldots ,a_M^i)$, generated from the PDE fields via FMM, ASC and FIM, forming $\sum _{j=1}^M a_j^i R_j$. This weighted result is then mapped back to the spatial domain by inverse FFT. In the pointwise branch, $\nu _{l-1}$ is processed by a pointwise weight matrix *W*. Finally, the inverse-FFT output and the pointwise output are summed together with a residual connection from $\nu _{l-1}$ to produce $\nu _l$. In the full SPINO version, green and red arrows denote the processing paths of blocks 1–3 and block 4, respectively. In the SPINO w/o FIM version, the FIM component is removed, and the corresponding weights $(a_1^i, \ldots , a_M^i)$ are generated via the green path for all four blocks; the red path is no longer used. (c) Feature mixture module (FMM). This module refines the input PDE fields at two time steps, $\mathbf {u}^i_{k-t,t}[\Omega ]$ and $\mathbf {u}^i_{k,t}[\Omega ]$, and encodes their SPIs using the PI encoder [37]. (d) Adjacent state connector (ASC). The ASC updates the hidden state $h_t$ by combining the current input $x_t$ with the previous hidden state $h_{t-1}$ through gating mechanisms (update and reset gates), which control information flow and memory. (e) Feature interaction module (FIM). The FIM enhances feature fusion by combining high-dimensional inputs $\nu _{l-1}$ with SPIs via self-attention and a multi-layer perceptron (MLP), producing the weights $(a_1^i, \ldots , a_M^i)$ for adaptive feature mixing.

We illustrate the main prediction workflow of SPINO in Fig. 2a. The input PDE fields ${\bf u}^i_{k,t}[\Omega ]$ are first encoded into a latent representation $v_0$ by the neural network *P*, processed through four SPINO blocks and finally decoded by the neural network *Q* to produce the prediction ${\bf u}^i_{k+t,t}[\Omega ]$. This prediction is then fed back into the network as a new input, allowing the process to be repeated recursively. Through this auto-regressive mechanism, SPINO can generate PDE solutions at arbitrary future time steps.

A key feature of SPINO is the use of SPIs, which are generated by the PI encoder $\mathcal {P}$ from the current input ${\bf u}^i_{k,t}[\Omega ]$ and continuously evolve during prediction, thereby providing dynamic physical guidance throughout the process. To enrich the temporal information encoded in SPIs, the adjacent state connector (ASC; Fig. 2d) integrates both past and current physical invariants, constructing more comprehensive sequential features and enhancing the model’s generalization capability. While SPIs improve model adaptability and generalization across prediction phases, their quality can be affected by noisy intermediate outputs, leading to accumulated errors. The proposed FMM (Fig. 2c) addresses this by employing a refinement module $\mathcal {D}$ to enhance SPIs, thereby boosting the operator’s robustness to noise. Finally, to compensate for the reduced influence of SPIs in deeper layers, the FIM (Fig. 2e) uses a self-attention mechanism in the final SPINO block to facilitate interaction between SPIs and high-level features, reinforcing physical guidance at deeper stages.

These core components—ASC, FMM and FIM—are seamlessly embedded into the structure of each SPINO block, as illustrated in Fig. 2b. Within a single block, the input feature $v_{l-1}$ is first transformed via the fast Fourier transform (FFT) to extract high-dimensional representations. To guide the network with physical prior knowledge, attention weights are generated from PDE solution fields through a pipeline consisting of FMM, ASC and an MLP, and are finally fused by FIM. These attention weights are then used to scale the spectral components across multiple branches, followed by a weighted summation to produce the output feature $v_l$. The aggregated result is transformed back to the spatial domain using an inverse FFT and added to the original input through a residual connection to produce the output $\nu _l$. Through the coordinated roles of ASC, FMM and FIM, SPINO effectively captures temporal dynamics and mitigates error accumulation in PDE prediction.

### Definition of physical invariants

In multi-physical scenarios, high-level physical information $\theta ^{i}$ reveals the underlying characteristics of a PDE, enabling clear differentiation among various types. MPNNs [41] show that incorporating the indicator of this physical information can substantially improve the generalization capabilities of neural networks. However, collecting such physical information can be infeasible or prohibitively expensive in many real-world applications. To address this limitation, PIANO [37] proposes using contrastive learning [42] to learn high-level representations of $\theta ^{i}$ from the input raw PDE fields $\mathrm{{\bf u}}^{i}_{k,t}$, as


(3) \begin{eqnarray*}
\mathcal {P}: \mathbb {R}^{n \times t} \rightarrow \mathbb {R}^{m}, \quad \mathcal {P}(\mathrm{{\bf u}}^{i}_{k,t}) = \mathbf {e}^{i},
\end{eqnarray*}

where $\mathrm{{\bf u}}^{i}_{k,t} \in \mathbb {R}^{n \times t}$ denotes the sequence of *t* consecutive PDE fields for the *i*th equation instance, *n* is the number of spatial discretization points, *m* is the dimension of the encoded representation, $\mathbf {e}^{i}$ represents the physical invariants and $\mathcal {P}$ is a parametric network referred to as the PI encoder. In PIANO, the PI is extracted from the initial frames of the PDE fields by applying $\mathcal {P}$, which may cause the fixed representation to become increasingly misaligned with the evolving system states during long-term prediction. Detailed descriptions of the PI encoder are provided in Section S3.

### The sliding physical invariants

We propose SPIs based on two intuitive thoughts. (1) For predicting $\mathrm{{\bf u}}_{2t,t}$, the extracted $\mathcal {P} (\tilde{\mathrm{{\bf u}}}_{t,t})$ better captures the current evolution trend of the PDE system compared with $\mathcal {P} (\mathrm{{\bf u}}_{0,t})$. (2) Incorporating both $\mathcal {P} (\tilde{\mathrm{{\bf u}}}_{t,t})$ and $\mathcal {P} (\mathrm{{\bf u}}_{0,t})$ increases the range of available PIs, allowing the model to capture more comprehensive dynamical patterns and improving the generalization of predictions. Inspired by these considerations, we formulate the SPINO framework for predicting $\mathrm{{\bf u}}_{k+t,t},\, k \ge t$, as


(4) \begin{eqnarray*}
\left\{ {\begin{array}{l}
\tilde{\mathbf {e}} = \mathrm{ASC}(\mathcal {P} (\mathrm{\tilde{{\bf u}}}_{k-t,t}), \mathcal {P} (\mathrm{\tilde{{\bf u}}}_{k,t})),\\
\mathrm{\tilde{{\bf u}}}_{k+t,t} = \mathcal {G}(\mathrm{\tilde{{\bf u}}}_{k,t}, \tilde{\mathbf {e}}),
\end{array}} \right.
\end{eqnarray*}

where ASC denotes the adjacent state connector for linking adjacent PIs and $\mathcal {G}$ is the neural operator that receives the SPI $\tilde{\mathbf {e}}$ and the input PDE field $\mathrm{\tilde{{\bf u}}}_{k,t}$. In this notation, all variables marked with a tilde (e.g. $\mathrm{\tilde{{\bf u}}}_{k,t}$, $\mathrm{\tilde{{\bf u}}}_{k\pm t,t}$ and $\tilde{\mathbf {e}}$) represent predicted quantities generated by the model rather than ground-truth values. In the case of $k = 0$, we define $\mathbf {e} = \mathcal {P} (\mathrm{{\bf u}}_{0,t})$. By incorporating SPIs $\tilde{\mathbf {e}}$ into the neural operator $\mathcal {G}$, the model gains the ability to capture long-term temporal dependencies, which are essential for stable and consistent PDE forecasting. Overall, the SPIs enable the model to continuously update the set of physical invariants during forecasting, thereby capturing the evolving dynamics of PDE systems more comprehensively. In parallel, the ASC provides a principled mechanism for propagating information between adjacent SPIs, enhancing temporal modeling. Together, these two components act synergistically: the SPIs contribute dynamic physical guidance, while the ASC captures and propagates temporal dependencies, leading to accurate long-term PDE prediction. See the Method section below for more details on the ASC.

### Feature mixture module

SPIs enable the model to learn distinct parameters for different equations across prediction phases. This improves time-dependent modeling, enhances generalization across systems and promotes consistency with physical information, thereby strengthening the robustness and accuracy of operator-learning frameworks. However, as shown in Equation (4), noisy predictions $\tilde{{\bf u}}_{k-t,t}$ and $\tilde{{\bf u}}_{k,t}$ can degrade the quality of the generated SPIs $\tilde{{\bf e}}$. These inferior SPIs, in turn, affect the prediction of ${\bf u}_{k+t,t}$, leading to a phenomenon known as error accumulation, which is particularly problematic in long-term prediction tasks. To address this, we propose the FMM, which mitigates the issue in two ways: by enhancing the quality of SPI generation and by improving the robustness of $\mathcal {G}$ to noisy SPIs.

*Enhancing the generation quality of SPIs.* The main reason for inferior SPIs is that the pretrained PI encoder $\mathcal {P}$ is sensitive to the predicted input $\tilde{\mathbf {u}}_{k,t}$, since it has been trained exclusively on exact solutions $\mathbf {u}_{k,t}$. This mismatch reduces it robustness under distribution shifts, where accumulated prediction inaccuracies (referred to as ‘noise’ in this work) may degrade its performance. To address this issue, we employ a refinement module $\mathcal {D}$ based on convolutional neural networks (CNNs). The role of $\mathcal {D}$ is to reduce these prediction errors and stabilize the representations extracted by $\mathcal {P}$, thereby improving the encoder’s robustness. Specifically, we modify Equation (4) as


(5) \begin{eqnarray*}
\left\{ {\begin{array}{l}
(\hat{\mathbf {u}}_{k-t,t},\, \hat{\mathbf {u}}_{k,t})= (\mathcal {D}(\tilde{\mathbf {u}}_{k-t,t}),\, \mathcal {D}(\tilde{\mathbf {u}}_{k,t})),\\
\tilde{\mathbf {e}} = \mathrm{ASC}(\mathcal {P}(\hat{\mathbf {u}}_{k-t,t}),\, \mathcal {P}(\hat{\mathbf {u}}_{k,t})),\\
\tilde{\mathbf {u}}_{k+t,t} = \mathcal {G}(\tilde{\mathbf {u}}_{k,t},\, \tilde{\mathbf {e}}),
\end{array}} \right.\end{eqnarray*}

where $\mathcal {D}$ is supervised by the mean-squared error loss between the exact solution ${\bf u}$ and the refined solution $\hat{{\bf u}}$.

*Improving the robustness of $\mathcal {G}$.* A basic training pipeline for learning the neural operator $\mathcal {G}$ is


\begin{eqnarray*}
{{\bf u}}_{k-t,t}\ {\stackrel{\longmapsto}{\mathbf {e}}}\ {\tilde{{\bf u}}}_{k,t}\ {\stackrel{\longmapsto }{\mathbf {\tilde{e}}}}\ {\tilde{{\bf u}}}_{k+t,t},
\end{eqnarray*}

where $\mathbf {e}$ and $\mathbf {\tilde{e}}$ are derived from respective PDE fields, with $\mathbf {e}$ representing the ground-truth features and $\mathbf {\tilde{e}}$ denoting the predicted ones. In this framework, $\mathcal {G}$ learns two extreme conditions: (1) both input PDE fields and SPIs are exact, and (2) both input PDE fields and SPIs are noisy. However, this process is difficult to optimize because the model exhibits limited capability at the beginning of training. As a result, the predicted $\mathrm{\tilde{{\bf u}}}_{k,t}$ substantially deviates from the true solution, and learning the mapping


\begin{eqnarray*}
\mathrm{\tilde{{\bf u}}}_{k,t}\ {\stackrel{\longmapsto }{\mathbf {\tilde{e}}}}\ \mathrm{\tilde{{\bf u}}}_{k+t,t}
\end{eqnarray*}

becomes ineffective and may even be harmful. To enhance the robustness and generalization of $\mathcal {G}$, we mix exact and noisy SPIs during training, making it challenging for the model to distinguish between real and perturbed SPIs. Specifically, a stratified batch-sampling strategy is employed, where each mini- batch contains samples drawn from two different predictive pipelines in fixed proportions:


(6) \begin{eqnarray*}
\left\{ {\begin{array}{c}\mathrm{{\bf u}}_{k-t,t}\ {\stackrel{\longmapsto } {\mathbf {e}}}\ \mathrm{\tilde{{\bf u}}}_{k,t}\ {\stackrel{\longmapsto }{\mathbf {\tilde{e}}}}\ \mathrm{\tilde{{\bf u}}}_{k+t,t}, \\
\mathrm{{\bf u}}_{k-t,t}\ {\stackrel{\longmapsto } {\mathbf {e}}}\ \mathrm{\tilde{{\bf u}}}_{k,t}\ {\stackrel{\longmapsto } {\mathbf {e}}}\ \mathrm{\tilde{{\bf u}}}_{k+t,t}. \end{array}}\right.
\end{eqnarray*}

See the Method section below for more details on the stratified batch-sampling strategy.

### Sliding physical invariant neural operator

We now introduce the complete workflow of our SPINO for solving time-dependent PDE systems. The fundamental mechanism of SPINO involves using the *t*-frame PDE fields ${\bf u}_{k,t}$ and the corresponding SPI ${\bf e}$ to predict the next *t*-frame PDE fields ${\bf u}_{k+t,t}$. This process is iterated until the target time *T* is reached. The core component of SPINO is a four-block FNO [24], which restricts the integral operator $\mathcal {K}$ to a mode-wise linear transformation in each layer. Specifically, the FFT is used to compute $\mathcal {K}$ efficiently. The Fourier convolution operator is defined as


(7) \begin{eqnarray*}
\mathcal {K}(\mathbf {v}_l) := \mathcal {F}^{-1}(\mathbf {R}\cdot \mathcal {F}(\mathbf {v}_l)),
\end{eqnarray*}

where $\mathbf {R}$ performs mode-wise multiplication in the Fourier domain and is applied only to the lower modes, $\mathbf {v}_l$ is the intermediate representation at the *l*th layer, and $\mathcal {F}$ and $\mathcal {F}^{-1}$ denote the FFT and its inverse. Consequently, the features in each layer are computed as


(8) \begin{eqnarray*}
\mathbf {v}_{l+1}=\sigma (\mathbf {W}\mathbf {v}_l + \mathcal {K}(\mathbf {v}_l)),
\end{eqnarray*}

where $\mathbf {W}\mathbf {v}_l$ represents the pointwise weight matrix *W* for the linear transformation acting on $\mathbf {v}_l$, and $\sigma$ is a non-linear activation function.

In SPINO, starting with the initial PDE fields $\mathrm{{\bf u}}_{0,t}$, the model first maps them into a higher-dimensional representation $\mathbf {v}_0$ via an MLP. Simultaneously, the SPIs ${\bf e}$ are extracted from $\mathrm{{\bf u}}_{0,t}$ using the pretrained PI encoder $\mathcal {P}$. To make the model physics-aware, we adopt the DyConv [40] technique to incorporate the SPIs into the neural operator $\mathcal {G}$. Specifically, the matrix $\mathbf {R}$ is expanded into *M* convolutional matrices of the same size, with the SPIs $\mathbf {e}$ regulating the integrated weights of each matrix:


(9) \begin{eqnarray*}
\mathbf {a} = \mathrm{SoftMax}(\mathrm{MLP}(\mathbf {e})) \in \mathbb {R}^M,\quad \mathbf {R} = \sum _{j=1}^{M} \mathbf {a}_j \mathbf {R}_j ,
\end{eqnarray*}

where the MLP transforms the SPIs $\mathbf {e}$ into *M* non-negative coefficients, and the SoftMax function normalizes them into a probability distribution $\mathbf {a}$.

In the above process, the SPI serves as a high-level physical prior that guides the earlier SPINO blocks. At these stages, the extracted representations are expected to primarily capture broader, less specialized structures—a behavior broadly observed in hierarchical neural architectures. Therefore, allowing the SPI to provide strong structural constraints is beneficial. As the network goes deeper, the learned features become increasingly specialized, encoding richer spatial-temporal patterns. In such cases, relying solely on the original SPI may not sufficiently align with the refined representations learned by the model. This motivates us to consider direct interaction between SPIs and the final-block features, enabling the physical priors to adaptively align with the most accurate representations produced by the network.

To achieve this, we propose the FIM, which incorporates a self-attention (SA) mechanism into the final SPINO block to facilitate information exchange between SPIs $\mathbf {e}$ and high-level features $\mathbf {v}_{l-1}$. Specifically, before the interaction, the features $\mathbf {v}_{l-1}$ are passed through a downsampling module to ensure channel alignment with $\mathbf {e}$. They are then concatenated along the sequence dimension:


(10) \begin{eqnarray*}
\mathbf {Z} = [\mathbf {e}; \mathrm{Down}(\mathbf {v}_{l-1}) ] \in \mathbb {R}^{(L_e+L_x)\times d},
\end{eqnarray*}

where $\mathbf {e}$ denotes the SPI sequence of length $L_e$ and $\mathrm{Down}(\mathbf {v}_{l-1})$ is the downsampled feature sequence of length $L_x$, both with embedding dimension *d*. Given $\mathbf {Z}$, the self-attention mechanism computes


(11) \begin{eqnarray*}
\left\{ {\begin{array}{l}
\mathbf {Q} = \mathbf {Z}\mathbf {W}^Q,\\
\mathbf {K} = \mathbf {Z}\mathbf {W}^K,\\
\mathbf {V} = \mathbf {Z}\mathbf {W}^V, \\
{\mathrm{SA}}(\mathbf {Z}) = {\mathrm{SoftMax}} \left(\frac{\mathbf {Q}\mathbf {K}^\top }{\sqrt{d}}\right)\mathbf {V}.
\end{array}} \right.
\end{eqnarray*}

This mechanism measures pairwise dependencies between $\mathbf {e}$ and the downsampled features, enabling the model to dynamically emphasize the most informative components and capture long-range correlations across different scales. Compared with static concatenation or linear mapping, this adaptive process allows FIM to better align the physical priors embedded in SPIs with the data-driven representations from $\mathbf {v}_{l-1}$, thus enhancing both expressiveness and physical consistency.

Based on the above design, the formulation for computing $\mathbf {a}$ is given as


(12) \begin{eqnarray*}
\left\{ {\begin{array}{l}
\left(\mathbf {e_{att}}, \mathbf {v}_{l-1}^{att}\right) = \mathrm{SA}(\mathbf {e}, \mathrm{Down}({\mathbf {v}_{l-1}})),\\
\mathbf {a}^{(e)} = {\rm {SoftMax}} ({\rm {MLP}}(\mathbf {e})),\\
\mathbf {a}^{(att)} = {\rm SoftMax}({\rm MLP}(\mathbf {e_{att}})),\\
\mathbf {a} = \mathbf {a}^{(e)} + \\lesssimmbda \mathbf {a}^{(att)},
\end{array}} \right.
\end{eqnarray*}

where $\\lesssimmbda$ is a hyperparameter that controls the influence of the second item on $\mathbf {a}$. This adjustment ensures that the learned attention weights adaptively integrate both the original SPIs $\mathbf {e}$ and the refined information from the SPINO block features $\mathbf {v}_{l-1}$, thereby bridging the gap between physics-inspired representations and high-level data-driven features. See the Method section below for more details on the SPINO loss function. A theoretical analysis of SPINO is provided in Section S8.

## EXPERIMENTS

### Datasets and implementation details

To comprehensively evaluate the scalability and effectiveness of our method, we select multiple datasets from different fields. These include two variants of the 1D Burgers’ equation, the 1D convection-diffusion equation, the 2D Navier–Stokes equation and the 2D Kolmogorov flow. The ground-truth data for 1D problems are generated using the Python package py-pde [43] with a fixed step size of $10^{-4}$. The final time *T* is set to 5 for the training set and 6 for the test set. For the 2D problems, the data are generated using the pseudo-spectral method with a time step of $10^{-4}$ and a $256\times 256$ grid. The data are then downsampled to a $64\times 64$ grid to match the settings in FNO [24]. The final times *T* are 20 and 24 for the training and test sets, respectively. Detailed implementation descriptions are provided in Section S2.

*Experiment E1: Burgers’ equation with varying external forces *f*.* Burgers’ equation is commonly used to model the nonlinear dynamics of various fluid dynamics systems. The one-dimensional Burgers’ equation with varying external forces *f* is given by


(13) \begin{eqnarray*}
\frac{\partial u}{\partial t} &=& - u \frac{\partial u}{\partial x} + 0.1 \Delta u + 0.1f(x),\nonumber\\
&& x \in [-\pi , \pi ], \quad u(\pm \pi , t) = 0,
\end{eqnarray*}

where $f(x)$ is a smooth function representing the external force. In this experiment, we evaluate the performance of SPINO and other baseline methods under 14 different external forces, uniformly sampled from the set $\lbrace 0, 1, \cos {(x)}, \cos {(2x)}, \cos {(3x)}, \sin {(x)}, \sin {(2x)},\\ \sin {(3x)}, \, \pm \tanh {(x)}, \pm \tanh {(2x)}, \pm \tanh {(3x)}\rbrace$.

*Experiment E2: Burgers’ equation with varying diffusivities *D*.* We simulate the 1D Burgers’ equation with spatially varying diffusivities, defined as


(14) \begin{eqnarray*}
\frac{\partial u}{\partial t} &=& - u \frac{\partial u}{\partial x} + 0.1 \nabla (D(x) \cdot \nabla u),\nonumber \\
&& x \in [-\pi , \pi ], \quad u(\pm \pi , t) = 0,
\end{eqnarray*}

where $D(x)$ is a smooth, non-negative function representing the spatially varying diffusivity. In this experiment, we evaluate the performance of SPINO and baseline methods under varying spatial fields using 10 diffusivity functions, uniformly sampled from the set $\lbrace 1, 2, 1 \pm \cos {(x)}, 1 \pm \sin {(x)} , 1 \pm \cos {(2x)}, 1 \pm \sin {(2x)} \rbrace$.

*Experiment E3: convection-diffusion equation (CDE) with varying boundary conditions $\mathcal {B}$.* We simulate 1D CDEs with varying boundary conditions, defined as


(15) \begin{eqnarray*}
\frac{\partial u}{\partial t} &=& 0.1 \Delta u + 0.1u + 0.1\sin {(2\pi x)},\nonumber\\
&& x \in [0, 1], \quad \mathcal {B}[u](x,t) = 0,
\end{eqnarray*}

where $\mathcal {B}[u](x, t) = 0$ represents the boundary conditions. In this experiment, we select four types of $\mathcal {B}$ to evaluate SPINO and other baseline methods under varying boundary conditions. The four boundary-condition types are the Dirichlet condition ($u = 0.2$), the Neumann condition ($\partial _{n} u = 0.2$), the curvature condition ($\partial _{n}^{2}u = 0.2$) and the Robin condition ($\partial _{n}u + u = 0.2$).

*Experiment E4: Navier–Stokes equation (NSE) with varying viscosity terms $\nu$.* We simulate the vorticity fields for 2D flows within a periodic domain $\Omega = [0, 1] \times [0, 1]$, governed by the NSEs:


(16) \begin{eqnarray*}
{\begin{array}{c}\displaystyle\frac{\partial \omega }{\partial t} = -(\mathbf {u} \cdot \nabla )\omega + \nu \Delta \omega + f(\mathbf {x}), \\
\omega = \nabla \times \mathbf {u}, \end{array}}
\end{eqnarray*}

where $f(\mathbf {x}) = 0.1 \sin {(2\pi ( \mathbf {x_1} + \mathbf {x_2} ))} + 0.1 \cos {(2\pi ( \mathbf {x_1} + \mathbf {x_2} ))}$ and $\nu \in \mathbb {R}^{+}$ represent the forcing function and viscosity term, respectively. The viscosity is a crucial component in NSEs, as it determines the level of turbulence in the flow. We generate NSE data with varying viscosity coefficients to simulate heterogeneity, ranging from $10^{-2}$ to $10^{-5}$. The viscosity fields become more complex as $\nu$ decreases because the nonlinear term $-(\mathbf {u} \cdot \nabla )\omega$ increasingly dominates the fluid motion.

*Experiment E5: Kolmogorov flow with varying viscosity terms $\nu$.* We simulate the vorticity fields for 2D NSEs within a periodic domain $\Omega =[0, 1] \times [0, 1]$, driven by Kolmogorov forcing [44]:


(17) \begin{eqnarray*}
\frac{\partial \omega }{\partial t} &=& -(\mathbf {u} \cdot \nabla )\omega + \nu \Delta \omega + 0.1\cos {(8\pi \mathbf {x_1})},\nonumber\\
\omega &=& \nabla \times \mathbf {u}.
\end{eqnarray*}

The fluid fields in Equation (17) produce more complex trajectories due to the Kolmogorov forcing. We generate NSE data with varying viscosity coefficients to simulate heterogeneity, ranging from $10^{-2}$ to $10^{-4}$.

### Baselines

We compare SPINO with five advanced baselines to demonstrate its superior performance in terms of prediction accuracy and generalization capability.

- FNO [24]: an effective operator that approximates the integral operator of the kernel in the frequency domain using the Fourier transform.
- U-net [45]: a convolutional neural network designed for semantic segmentation, featuring an encoder-decoder structure with skip connections for precise localization and efficient feature extraction. Recently, it has become popular as a surrogate model for solving PDEs.
- Low-rank decomposition network (LordNet) [46]: a convolutional neural PDE solver that incorporates a low-rank decomposition layer to extract the primary patterns.
- MultiWaveleT (MWT)-based model [47]: a neural operator that compresses the kernel of the corresponding operator using fine-grained wavelets.
- PIANO [37]: a framework for operator learning that decodes physical-invariant information from PDE fields with varying physical dynamics and incorporates it into neural operators for prediction tasks.

### Comparison with the state of the art

#### Quantitative results

Table 1 systematically compares SPINO with state-of-the-art PDE solvers across five equations, evaluating both prediction accuracy ($E_{\ell _{2}}(\%)$, $E_{\ell _{\infty }}(\%)$) and computational efficiency. Our method demonstrates consistent superiority over all baselines, particularly surpassing PIANO with error reductions of $34.3\%$–$79.8\%$ in the training domain and $7.7\%$–$76.5\%$ in the future domain. SPINO achieves superior performance by leveraging its unique capacity to adaptively learn phase-specific invariants, as shown in Equation (5). However, this enhanced predictive accuracy is achieved at the expense of significantly increased computational cost. The model complexity results in parameter counts of 1.118 million for 1D problems and 11.873 million for 2D problems. Consequently, SPINO incurs training times that are 2 to 7 times longer and inference times that are 2 to 15 times longer than those of the state-of-the-art methods, although inference remains within seconds. Further discussion of the trade-off between model complexity and predictive accuracy is provided in Section S4.1. A more controlled evaluation accounting for hardware differences between PIANO+FNO and SPINO is provided in Section S11.

**Table 1.** Results of the PDE simulations for experiments E1–E5.

		Training domain		Future domain		Time per epoch (s)		Param
Data	Model	$E_{\ell _{2}}(\%)$	$E_{\ell _{\infty }}(\%)$	$E_{\ell _{2}}(\%)$	$E_{\ell _{\infty }}(\%)$	Train (s)	Infer (s)	#(million)
E1. Burgers’ equation	FNO	$0.669_{\pm \ 0.124}$	$0.978_{\pm \ 0.029}$	$1.062_{\pm \ 0.039}$	$1.340_{\pm \ 0.158}$	0.128	0.018	0.757
with varying external	LordNet	$1.660_{\pm \ 0.058}$	$2.406_{\pm \ 0.262}$	$2.782_{\pm \ 0.111}$	$3.529_{\pm \ 0.213}$	0.317	0.138	0.810
forces *f*	MWT	$1.962_{\pm \ 0.250}$	$2.737_{\pm \ 0.450}$	$2.764_{\pm \ 0.379}$	$3.572_{\pm \ 0.514}$	0.460	0.111	0.789
	Unet	$2.576_{\pm \ 0.124}$	$4.205_{\pm \ 0.108}$	$3.280_{\pm \ 0.084}$	$4.687_{\pm \ 0.158}$	0.256	0.041	0.860
	PIANO+FNO	$0.492_{\pm \ 0.045}$	$0.611_{\pm \ 0.045}$	$0.536_{\pm \ 0.046}$	$0.700_{\pm \ 0.038}$	0.147	0.022	0.762
	PIANO+Unet	$1.605_{\pm \ 0.264}$	$3.130_{\pm \ 0.685}$	$1.796_{\pm \ 0.386}$	$2.946_{\pm \ 0.526}$	0.299	0.039	0.766
	SPINO*	$\underline{0.286_{\pm \ 0.070}}$	$\underline{0.452_{\pm \ 0.075}}$	$\underline{0.325_{\pm \ 0.073}}$	$\underline{0.513_{\pm \ 0.094}}$	0.706	0.075	1.118
	SPINO w/o FIM*	$\mathbf {0.227_{\pm \ 0.003}}$	$\mathbf {0.361_{\pm \ 0.001}}$	$\mathbf {0.244_{\pm \ 0.005}}$	$\mathbf {0.403_{\pm \ 0.004}}$	0.535	0.052	0.976
E2. Burgers’ equation	FNO	$6.328_{\pm \ 0.162}$	$10.847_{\pm \ 0.251}$	$13.111_{\pm \ 0.384}$	$19.379_{\pm \ 0.649}$	0.128	0.018	0.757
with varying	LordNet	$8.471_{\pm \ 0.628}$	$22.016_{\pm \ 6.849}$	$23.786_{\pm \ 7.989}$	$62.977_{\pm \ 35.304}$	0.317	0.138	0.810
diffusivities *D*	MWT	$6.381_{\pm \ 0.069}$	$12.355_{\pm \ 0.580}$	$12.013_{\pm \ 0.266}$	$18.952_{\pm \ 1.082}$	0.460	0.111	0.789
	Unet	$7.087_{\pm \ 1.680}$	$12.592_{\pm \ 2.750}$	$13.593_{\pm \ 3.413}$	$20.221_{\pm \ 5.280}$	0.256	0.041	0.860
	PIANO+FNO	$4.559_{\pm \ 0.092}$	$8.932_{\pm \ 0.312}$	$8.421_{\pm \ 0.440}$	$13.680_{\pm \ 1.174}$	0.147	0.022	0.762
	PIANO+Unet	$4.149_{\pm \ 0.985}$	$8.879_{\pm \ 1.106}$	$7.342_{\pm \ 2.072}$	$12.330_{\pm \ 3.015}$	0.299	0.039	0.766
	SPINO*	$\mathbf {2.996_{\pm \ 0.113}}$	$\mathbf {3.177_{\pm \ 0.060}}$	$\mathbf {6.682_{\pm \ 0.223}}$	$\mathbf {9.128_{\pm \ 0.478}}$	0.329	0.089	1.118
	SPINO w/o FIM*	$\underline{3.699_{\pm \ 0.101}}$	$\underline{8.136_{\pm \ 0.289}}$	$\underline{6.902_{\pm \ 0.243}}$	$\underline{11.429_{\pm \ 0.368}}$	0.321	0.078	0.976
E3. CDE with varying	FNO	$1.127_{\pm \ 0.256}$	$1.742_{\pm \ 0.346}$	$1.468_{\pm \ 0.394}$	$2.041_{\pm \ 0.420}$	0.128	0.018	0.757
boundary conditions $\mathcal {B}$	LordNet	$0.605_{\pm \ 0.039}$	$0.990_{\pm \ 0.048}$	$0.901_{\pm \ 0.072}$	$0.832_{\pm \ 0.063}$	0.317	0.138	0.810
	MWT	$0.662_{\pm \ 0.037}$	$1.232_{\pm \ 0.107}$	$0.781_{\pm \ 0.113}$	$1.385_{\pm \ 0.148}$	0.460	0.111	0.789
	Unet	$12.565_{\pm \ 1.752}$	$20.786_{\pm \ 2.976}$	$20.335_{\pm \ 3.100}$	$22.686_{\pm \ 3.511}$	0.256	0.041	0.860
	PIANO+FNO	$0.416_{\pm \ 0.180}$	$0.893_{\pm \ 0.338}$	$0.708_{\pm \ 0.403}$	$1.098_{\pm \ 0.547}$	0.148	0.022	0.763
	PIANO+Unet	$2.921_{\pm \ 0.363}$	$5.773_{\pm \ 0.767}$	$3.611_{\pm \ 0.830}$	$5.446_{\pm \ 0.676}$	0.299	0.039	0.767
	SPINO*	$\mathbf {0.147_{\pm \ 0.017}}$	$\mathbf {0.338_{\pm \ 0.029}}$	$\underline{0.377_{\pm \ 0.043}}$	$\underline{0.555_{\pm \ 0.092}}$	0.575	0.080	1.118
	SPINO w/o FIM*	$\underline{0.161_{\pm \ 0.003}}$	$\underline{0.346_{\pm \ 0.002}}$	$\mathbf {0.236_{\pm \ 0.020}}$	$\mathbf {0.492_{\pm \ 0.068}}$	0.472	0.067	0.976
E4. NSE with varying	FNO	$10.433_{\pm \ 0.298}$	$16.937_{\pm \ 0.302}$	$30.702_{\pm \ 1.043}$	$56.563_{\pm \ 0.949}$	0.384	0.182	2.085
viscosity terms $\nu$	LordNet	$8.469_{\pm \ 0.633}$	$15.574_{\pm \ 0.863}$	$30.348_{\pm \ 0.838}$	$57.728_{\pm \ 1.514}$	1.031	0.547	2.069
	MWT	$10.135_{\pm \ 0.346}$	$17.917_{\pm \ 0.253}$	$32.232_{\pm \ 0.713}$	$61.572_{\pm \ 1.487}$	1.067	0.229	2.295
	Unet	$9.054_{\pm \ 0.204}$	$18.483_{\pm \ 0.381}$	$31.830_{\pm \ 0.496}$	$60.106_{\pm \ 0.299}$	0.335	0.089	3.038
	PIANO+FNO	$4.652_{\pm \ 0.396}$	$9.191_{\pm \ 0.605}$	$17.393_{\pm \ 0.672}$	$39.953_{\pm \ 1.107}$	0.395	0.138	2.020
	PIANO+Unet	$6.070_{\pm \ 0.397}$	$15.356_{\pm \ 0.914}$	$20.132_{\pm \ 1.288}$	$47.079_{\pm \ 2.144}$	0.440	0.111	1.941
	SPINO*	$\mathbf {2.904_{\pm \ 0.002}}$	$\mathbf {5.797_{\pm \ 0.024}}$	$\mathbf {16.048_{\pm \ 0.073}}$	$\mathbf {36.508_{\pm \ 0.215}}$	2.471	2.048	11.873
	SPINO w/o FIM*	$\underline{3.377_{\pm \ 0.080}}$	$\underline{6.801_{\pm \ 0.086}}$	$\underline{17.260_{\pm \ 0.174}}$	$\underline{39.441_{\pm \ 0.764}}$	2.416	2.021	2.739
E5. Kolmogorov flow	FNO	$4.017_{\pm \ 0.101}$	$5.250_{\pm \ 0.171}$	$5.241_{\pm \ 0.027}$	$6.842_{\pm \ 0.219}$	0.384	0.182	2.085
with varying viscosity	LordNet	$6.559_{\pm \ 0.969}$	$8.159_{\pm \ 2.259}$	$11.343_{\pm \ 1.448}$	$17.940_{\pm \ 8.683}$	1.031	0.547	2.069
terms $\nu$	MWT	$4.663_{\pm \ 0.285}$	$5.769_{\pm \ 0.350}$	$6.511_{\pm \ 0.103}$	$8.062_{\pm \ 0.272}$	1.067	0.229	2.295
	Unet	$9.807_{\pm \ 2.673}$	$19.449_{\pm \ 6.144}$	$13.949_{\pm \ 3.593}$	$27.505_{\pm \ 9.510}$	0.335	0.089	3.038
	PIANO+FNO	$1.908_{\pm \ 0.074}$	$2.419_{\pm \ 0.040}$	$2.840_{\pm \ 0.126}$	$3.552_{\pm \ 0.126}$	0.395	0.138	2.020
	PIANO+Unet	$6.704_{\pm \ 0.201}$	$12.143_{\pm \ 0.119}$	$9.676_{\pm \ 0.248}$	$16.495_{\pm \ 0.168}$	0.440	0.111	1.941
	SPINO*	$\mathbf {0.385_{\pm \ 0.022}}$	$\mathbf {0.666_{\pm \ 0.039}}$	$\mathbf {0.665_{\pm \ 0.037}}$	$\mathbf {1.119_{\pm \ 0.058}}$	2.509	2.044	11.873
	SPINO w/o FIM*	$\underline{0.534_{\pm \ 0.004}}$	$\underline{0.824_{\pm \ 0.031}}$	$\underline{0.869_{\pm \ 0.003}}$	$\underline{1.317_{\pm \ 0.029}}$	2.469	1.936	2.739

Note that the ‘*’ mark indicates that the corresponding model was trained and evaluated on an NVIDIA V100 GPU, whereas baseline models without this mark were originally trained on an NVIDIA A100 GPU. The performance and training time results for these baseline models are taken directly from their original publications.

Here, $E_{l_2}(\%)$ and $E_{l_\infty }(\%)$ denote the relative $L_2$ and $L_{\infty }$ errors, respectively. The table also reports computational costs and parameter counts, with values for SPINO including the expenses of the neural operator. The best results for each task are highlighted in bold, and the suboptimal results are underlined.

To address this computational overhead and improve accessibility, we introduce a SPINO w/o FIM version that removes FIM while retaining the core SPI-based designs. The rationale for this choice is two-fold. First, FIM is specifically designed to enhance representation learning in high-dimensional and multi-scale PDE systems. In contrast, its contribution is less critical in relatively simple or low-dimensional problems. Second, FIM introduces a noticeable increase in parameter count, making it the heaviest among the added modules. Therefore, removing FIM provides a natural simplification while preserving the essential benefits of SPI and FMM. As shown in Table 1, this variant achieves a better trade-off between accuracy and efficiency, delivering competitive performance on most benchmarks with substantially fewer parameters and shorter training times. Presenting both versions allows practitioners to choose between maximum accuracy and a lightweight, easily deployable alternative according to application needs.

#### Qualitative results

**Figure 3.**
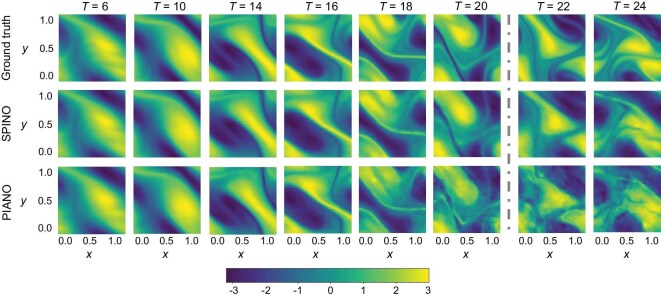
Comparison of the vorticity fields in the NSE between SPINO and PIANO from *T* = 6 to 24 in the periodic domain $[0, 1]^2$ for a 2D turbulent flow. Times $T = \lbrace 6, 10, 14, 16, 18, 20\rbrace$ correspond to the training domain, whereas $T = \lbrace 22, 24\rbrace$ correspond to the future domain.

To better analyze the temporal evolution of predicted solutions, we examine the vorticity fields in the NSE (E4) dataset over the time interval from $T = 6$ to $T = 24$, as visualized in Fig. 3. A comparison with the state-of-the-art PIANO method further highlights SPINO’s improved ability to capture long-term dynamics. Specifically, within the training domain, SPINO clearly outperforms PIANO by more effectively capturing the intricate details of fluid dynamics, demonstrating its superior ability to model the system’s complex behavior. This advantage underscores SPINO’s strength in interpolation tasks, where the data are available and the model can leverage learned patterns to make accurate predictions. However, in the future domain—where no supervised data are available for training—all methods face difficulties in producing precise vorticity-field predictions. Despite these challenges, SPINO continues to provide more accurate forecasts of the fluid dynamics trends compared with PIANO, demonstrating its enhanced capability for extrapolating from past observations. This performance improvement is directly attributable to the SPIs, which enable the model to capture the complex, evolving dynamics of physical systems more effectively. As a result, SPINO achieves substantial improvements in accuracy on both interpolation and extrapolation tasks.

### Ablation study

To explicitly analyze the contribution of each proposed component, we conduct a comprehensive ablation study on five datasets (E1–E5). We progressively introduce the SPI (with ASC), the FMM and the FIM, reporting the relative $L_2$ error for each configuration. Table 2 summarizes the results. A summary of the distinct roles of these components is provided at the end of this section.

**Table 2.** The overall ablation studies of the different components of our method on five datasets.

Components in SPINO			$E_{\ell _{2}}(\%)$ in training domain				
SPI	FMM	FIM	E1	E2	E3	E4	E5
			0.464	4.510	0.495	4.436	0.749
✔			0.443	4.009	0.422	3.740	0.546
		✔	0.314	4.149	0.160	3.823	0.488
✔	✔		**0.227**	3.699	0.161	3.377	0.534
✔		✔	0.372	3.384	0.732	3.321	0.552
✔	✔	✔	0.286	**2.996**	**0.147**	**2.904**	**0.385**

‘SPI’ denotes the PIs generated by the PI encoder from the current prediction; if SPI is not checked, a fixed PI is used instead. ‘FMM’ denotes the feature mixture module, and ‘FIM’ denotes the feature interaction module. Note that FMM is inherently dependent on SPI, so its ablation is conducted on top of SPI rather than in isolation. The best results for each task are highlighted in bold.

#### Ablation study on SPI

The SPI serves as the core module and the foundation for the other components of the framework, as both FMM and FIM rely on its dynamically updated invariant representations to perform their respective functions. Unlike the fixed PI, which is computed once from the initial condition and remains unchanged throughout the rollout, SPI recomputes invariants from the current PDE field and integrates them with historical invariants stored in the ASC. This design enables SPI to capture evolving dynamics and adapt to phase shifts during long-term forecasting, ensuring that the learned representation remains consistent with the underlying physical system.

As shown in Table 2, the error reductions range from 4.5% to 33.3%. This performance gain demonstrates that dynamically updated invariants are crucial for accurately modeling time-varying systems, whereas static PI representations become less informative as predictions progress. SPI therefore serves as the core module for delivering phase-specific guidance throughout auto-regressive rollouts. Further details on SPI are provided in Section S4.2.

#### Ablation study on FMM

The FMM is designed to improve both the quality of SPIs and the robustness of the operator $\mathcal {G}$, ultimately mitigating error accumulation during long-term auto-regressive predictions. Its primary function is two-fold: (i) a refinement module, $\mathcal {D}$, is employed to refine predicted fields before computing SPIs, ensuring that subsequent SPIs are derived from cleaner inputs; (ii) a stratified batch-sampling strategy is introduced to feed a mixture of clean and noisy SPIs to the operator $\mathcal {G}$, encouraging the model to learn SPIs that remain consistent even when the inputs are inaccurate.

As shown in the fifth and sixth rows of Table 2, the inclusion of FMM leads to a significant reduction in error, ranging from $11.4\%$ to $79.9\%$ across different experiments. This highlights its effectiveness in refining the SPI and enhancing the model’s predictive stability in noisy scenarios, ultimately mitigating long-term error accumulation. Further details of the FMM are provided in Section S4.3, and additional quantitative analyses illustrating error propagation over time, both with and without FMM, are presented in Section S5.

#### Ablation study on FIM

The FIM is used to facilitate the information exchange between SPIs and high-level features obtained from the last SPINO block. Building upon SPI, the addition of FIM further enhances performance, particularly in high-dimensional and multi-scale scenarios.

As shown in Table 2, the inclusion of FIM leads to performance improvements across the majority of experiments compared with models without FIM. Specifically, when comparing the first and third rows, as well as the fourth and sixth rows, the results exhibit noticeable gains in predictive accuracy. Error reductions range from approximately $8\%$ to $67.6\%$ in the first comparison and from $8.7\%$ to $27.9\%$ in the second, demonstrating the effectiveness of FIM in enhancing the model’s ability to capture relevant features and improve prediction performance. However, in relatively simple or low-frequency scenarios, such as E1 (Burgers’ equation), the inclusion of FIM slightly degrades performance. A plausible reason is that the attention-based interaction in FIM may introduce unnecessary feature mixing when the underlying dynamics are already effectively captured by SPI and FMM. In such cases, the additional attention weights may overemphasize minor variations or noise, leading to suboptimal generalization. This observation suggests that, while FIM enhances performance in complex, high-dimensional PDE systems, its benefits are less pronounced—and can even be detrimental—in simpler settings where less cross-feature integration is required. Further details of FIM are provided in Section S4.4.

#### Summary

The ablation study clearly identifies the source of performance improvements.

- SPI serves as the core module of the framework, enhancing long-term predictive accuracy by dynamically updating physical invariants to adapt to evolving dynamics and maintain physical consistency over time.
- FMM provides substantial gains in scenarios prone to error accumulation, stabilizing the predictive process and improving robustness.
- FIM provides additional improvements in high-dimensional and multi-scale tasks, further reducing prediction errors when combined with the other modules.

These results provide explicit evidence that each component plays a distinct and complementary role, and that the observed performance gains are directly attributable to their inclusion.

## CONCLUSIONS

To summarize, we have presented the formulation, implementation and effectiveness of SPINO, a novel neural operator framework that dynamically integrates physical invariants into PDE forecasting. SPIs are introduced to learn distinct model parameters for different equations across various prediction phases, enabling the model to adapt to evolving system dynamics. Through the integration of an ASC, SPINO captures long-term temporal dependencies among invariant features. Furthermore, the proposed FMM improves prediction stability by mitigating error accumulation through a refinement module and a stratified batch-sampling strategy, while the FIM enhances the interaction between SPIs and high-level features. Extensive experiments further verify the effectiveness of these designs, demonstrating SPINO’s superior generalization and long-term predictive performance across diverse PDE benchmarks.

While SPINO demonstrates strong performance, several challenges remain to be addressed in future work.

- *Extension to irregular and adaptive grids.* The current framework is primarily trained on structured, uniform grids. Achieving consistent training and inference on unstructured grids remains a challenging yet essential goal. Promising strategies include mapping irregular domains into latent uniform spaces (e.g. Geo-FNO [48]) or employing attention-based neural operators for irregular meshes (e.g. GNOT [31]). Integrating such techniques, or developing dedicated geometric encoders, represents a valuable step toward extending SPINO to fully irregular and adaptive meshes.
- *Scalability to higher-dimensional problems.* Extending SPINO from 2D to 3D domains introduces challenges such as increased computational cost, memory demand and high-dimensional operator representation. Future research will focus on improving scalability through domain decomposition, lightweight network design and efficient parameter-sharing strategies.
- *Application to real-world physical systems.* We aim to extend SPINO to real-world physical modeling tasks involving strong nonlinearity, limited data and realistic noise. A preliminary study on global sea surface temperature (SST) prediction, presented in Section S9, shows that SPINO effectively captures spatial-temporal dynamics and maintains long-term stability. Building on this study, our future work will extend SPINO to more challenging and diverse real-world scenarios, such as multi-variable climate and ocean dynamics and other data-scarce multi-physics systems, where stronger nonlinearity, higher dimensionality and limited data impose additional challenges for operator learning.
- *Generalization across spatial resolutions.* The current framework performs well at its trained grid scale but may experience accuracy loss on unseen resolutions. We plan to explore multi-resolution training, frequency alignment and adaptive operator designs to enhance robustness and extend applicability to real-world settings with variable spatial grids.

## METHOD

### Adjacent state connector

The ASC is a lightweight gating mechanism inspired by the gated recurrent unit (GRU) [49], designed to connect adjacent states within our framework. We specifically adopt GRU-inspired gating for temporal modeling because it strikes a balance between model expressiveness and efficiency. within, the relative advantages of GRU compared with conventional recurrent neural networks (RNNs) and long short-term memories (LSTMs) have been systematically investigated in prior studies [50], and our design choice is grounded in these well-established conclusions. Compared with a vanilla RNN, GRU-style gating mitigates vanishing-gradient issues and better preserves long-term dependencies. Compared with the more complex LSTM unit, GRU retains only the essential gating operations, thereby reducing redundant parameters and computational overhead. Furthermore, in contrast to transformer-based sequence models, which incur quadratic complexity in sequence length, the GRU provides a more efficient alternative, particularly suited for the local, step-to-step temporal connections in PDE forecasting. Building on these advantages, the ASC adopts the GRU gating structure but focuses exclusively on linking adjacent prediction states rather than maintaining long-term hidden memory. This design ensures efficient and stable information propagation between consecutive steps, making it particularly well suited for iterative PDE forecasting tasks. The detailed computation process of the GRU is defined as


(18) \begin{eqnarray*}
\left\{ {\begin{array}{l}
\mathbf {r} = \sigma (\mathbf {W_{ir}} \mathbf {x} + \mathbf {b_{ir}} + \mathbf {W_{hr}} \mathbf {h} + \mathbf {b_{hr}}),\\
\mathbf {z} = \sigma (\mathbf {W_{iz}} \mathbf {x} + \mathbf {b_{iz}} + \mathbf {W_{hz}} \mathbf {h} + \mathbf {b_{hz}}),\\
\mathbf {n} = \tanh (\mathbf {W_{in}} \mathbf {x} + \mathbf {b_{in}} + \mathbf {r}\,\, *\\ \qquad \mathbf {(W_{hn}} \mathbf {h} + \mathbf {b_{hn}})),\\
\tilde{\mathbf {e}} = (1 - \mathbf {z}) * \mathbf {n} + \mathbf {z} * \mathbf {h},
\end{array}} \right.
\end{eqnarray*}

where $\mathbf {x}$ denotes the current input, $\mathbf {h}$ denotes the previous hidden state, $\mathbf {W_{ir}}, \mathbf {W_{hr}}, \mathbf {W_{iz}}, \mathbf {W_{hz}}, \mathbf {W_{in}}, \mathbf {W_{hn}}$ are learnable matrices, $\mathbf {b_{ir}}, \mathbf {b_{hr}}, \mathbf {b_{iz}}, \mathbf {b_{hz}}, \mathbf {b_{in}}, \mathbf {b_{hn}}$ are learnable biases, $\sigma$ is the sigmoid function, $\tanh$ is the hyperbolic tangent function and $*$ denotes the Hadamard product. The final result, $\tilde{\mathbf {e}}$, integrates information from both $\mathbf {x}$ and $\mathbf {h}$, which correspond to $\mathcal {P} (\tilde{\mathrm{{\bf u}}}_{k,t})$ and $\mathcal {P} (\tilde{\mathrm{{\bf u}}}_{k-t,t})$ in Equation (4), respectively.

### Stratified batch-sampling strategy

Given a batch size of $B$, the number of samples assigned to each pipeline is computed as


(19) \begin{eqnarray*}
B_1 = \lfloor r_1(s) B \rfloor , \qquad B_2 = B - B_1,
\end{eqnarray*}

where $B_1$ and $B_2$ represent the number of training samples following each predictive scheme, ensuring that all samples within a batch contribute to diverse learning pathways.

Within each batch, the selected samples are processed through the following predictive pipelines:


(20) \begin{eqnarray*}
\left\{ {\begin{array}{c}\mathrm{{\bf u}}_{k-t,t}\ {\stackrel{\longmapsto } {\mathbf {e}}}\ \mathrm{\tilde{{\bf u}}}_{k,t}\ {\stackrel{\longmapsto }{\mathbf {\tilde{e}}}}\ \mathrm{\tilde{{\bf u}}}_{k+t,t} \quad \mathrm{for } B_1 \mathrm{ samples}, \\
\mathrm{{\bf u}}_{k-t,t}\ {\stackrel{\longmapsto }{\mathbf {e}}}\ \mathrm{\tilde{{\bf u}}}_{k,t}\ {\stackrel{\longmapsto }{\mathbf {e}}}\ \mathrm{\tilde{{\bf u}}}_{k+t,t} \quad \mathrm{for } B_2 \mathrm{ samples}. \end{array}}\right.
\end{eqnarray*}

The sampling ratios $r_1(s)$ and $r_2(s)$ are defined as


(21) \begin{eqnarray*}
\left\{ {\begin{array}{l}
r_1(s) = \frac{s}{\mathrm{epoch}},\\
r_2(s) = 1 - r_1(s),
\end{array}} \right.
\end{eqnarray*}

where $\mathrm{epoch}$ represents the total number of training epochs, and $s$ denotes the current training step. As training progresses, the batch composition gradually shifts, allowing the neural operator $\mathcal {G}$ to increasingly utilize more informative prediction pathways. This dynamic adjustment enhances the model’s ability to integrate subsequent information while maintaining robustness across different levels of noise.

### SPINO loss function

To effectively train the proposed SPINO, we design a loss function that jointly optimizes the neural operator $\mathcal {G}$ and the refinement module $\mathcal {D}$. The total loss comprises two components: the prediction loss from the neural operator and the reconstruction loss from the refinement module. Specifically, the loss is formulated as


(22) \begin{eqnarray*}
\mathcal {L} &=& \frac{\lambda_1}{N} \sum _{i=1}^{N} \sum _{\tau \in \lbrace k,\, k+t\rbrace } \Vert u_{\tau ,t}^i - \tilde{u}_{\tau ,t}^i \Vert ^2 \nonumber \\
&&\quad + \frac{\lambda_1 \lambda_2}{N} \sum _{i=1}^{N} \sum _{\tau \in \lbrace k,\, k+t\rbrace } \Vert u_{\tau ,t}^i - \hat{u}_{\tau ,t}^i \Vert ^2,\nonumber \\
\end{eqnarray*}

where $u_{\tau ,t}^i$ denotes the ground-truth solution for the *i*th sample at time index $\tau \in \lbrace k, k+t\rbrace$, while $\tilde{u}_{\tau ,t}^i$ and $\hat{u}_{\tau ,t}^i$ represent the outputs of the neural operator $\mathcal {G}$ and the refinement module $\mathcal {D}$, respectively.

The loss comprises a data-fidelity term and a refinement-regularization term. The former guides $\mathcal {G}$ to match predicted and true solutions, while the latter promotes consistency between refined outputs and ground truth. Hyperparameters $\\lesssimmbda _1$ and $\\lesssimmbda _2$ control their relative importance. This formulation encourages accurate long-term predictions and robustness to noise in the sliding physical invariants.

## Supplementary Material

nwag027_Supplemental_File
