# Supplementary material for: Sliding physical invariant neural operator for long-term prediction of complex dynamics in physical systems
Source: Natl Sci Rev. 2026 Jan 27;13(5):nwag027. doi: 10.1093/nsr/nwag027 (PMC13268997; doi:10.1093/nsr/nwag027)
Supplement: nwag027_Supplemental_File [file nwag027_supplemental_file.pdf]

Supplementary Materials for Sliding physical invariant neural operator for long-term prediction of complex dynamics in physical systems

Yanjie Wang[1], Ying Li[2,*], Yaxin Peng[3,*], and Shihui Ying[1,4,*]

[1]School of Mechanics and Engineering Science, Shanghai University, Shanghai 200444, China

[2]School of Computer Engineering and Science, Shanghai University, Shanghai 200444, China

[3]Department of Mathematics, School of Science, Shanghai University, Shanghai, 200444, China

[4]Shanghai Institute of Applied Mathematics and Mechanics, Shanghai University, Shanghai 200444, China

[*]**Corresponding author(s).** E-mail(s): yinglotus@shu.edu.cn; yaxin.peng@shu.edu.cn; shying@shu.edu.cn;

Contents

1 Background and motivation **4**

2 Experimental details **5**

2.1 Architecture of SPINO 5

2.2 Training details 6

2.3 Evaluation metric 6

3 Details of PI encoder **7**

3.1 Architecture of PI encoder 7

3.2 Learning strategy for PI encoder 7

3.3 PI encoder training details and efficiency 9

3.4 Ablation of PI encoder 10

4 More ablation on key modules **11**

4.1 Trade-off between model complexity and predictive accuracy 11

4.2 Ablation on Sliding Physical Invariants (SPI) 13

4.3 Ablation on Feature Mixture Module (FMM) 14

4.4 Ablation on Feature Interaction Module (FIM) 16

5 Quantitative analysis of error accumulation **17**

6 Extrapolation with progressive data availability **19**

7 Super-resolution capability of SPINO **20**

8 Theoretical analysis of SPINO: error propagation and stability in long-term predictions **22**

9 Evaluation of SPI-based operator learning in real-world scenario **31**

10 Other experiments **33**

10.1 1D Burgers' equation with a lower viscosity $\nu = 0.01$ 33

10.2 1D Burgers' equation with diverse initial conditions 35

10.3 1D Diffusion-sorption equation 37

10.4 2D Wave equation 40

10.5 2D Shallow water equation 41

11 Computational efficiency analysis **43**

11.1 Training time considerations 43

11.2 Inference efficiency analysis 43

1 Background and motivation

Operator learning aims to simulate the underlying physical system to solve an entire family of Partial Differential Equations (PDEs), rather than focusing on a single instance of the equation. To achieve this goal, current approaches encode the initial conditions of PDEs into physical invariants (PIs). These PIs are then used to guide the network in solving a broad range of PDEs. Although initial PIs effectively capture short-term properties of equations, they inherently fail to represent the intricate long-term evolution of solutions, leading to a decrease in predictive accuracy over extended temporal horizons.

Motivated by the evolving nature of time-dependent systems, we raise a natural question: is it possible to learn phase-specific parameters to improve the modeling of temporal dynamics across different PDEs? To that end, we propose the Sliding Physical Invariant Neural Operator (SPINO) which integrates the Sliding Physical Invariants (SPIs) into neural operators using dynamic convolutional (DyConv) kernels [1], aiming to achieve stable long-term predictions of system behaviors. Although the idea of learning SPIs is impressive, it also poses the risk of error accumulation. As shown in Supplementary Figure 1, while sliding clear PIs derived from true solutions enhance performance, sliding noisy PIs from predicted solutions may degrade subsequent predictions. To address this, we propose a Feature Mixture Module (FMM) to improve the quality of generated SPIs and strengthen the robustness of the neural operator.

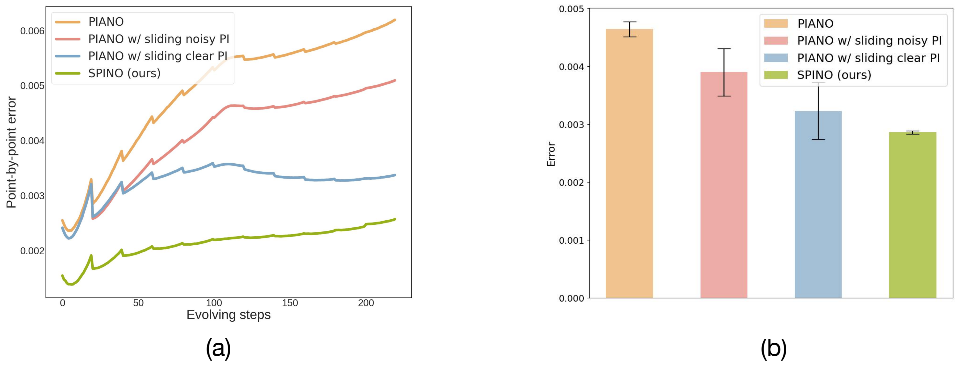

Supplementary Figure 1: Performance comparison in Burgers' equation among PIANO, PIANO with sliding noisy PIs, PIANO with sliding clear PIs, and our SPINO. (a) Point-by-point error from time step 0 to 219. Note that t = 0 to 179 corresponds to training domain, while t = 180 to 219 represents future domain. (b) The relative L_2 error over the entire time domain.

2 Experimental details

2.1 Architecture of SPINO

The SPINO architecture is composed of four stacked neural operator layers based on enhanced Fourier integral operators [2] integrated with three key components: Sliding Physical Invariants (SPIs), Feature Mixture Module (FMM), and Feature Interaction Module (FIM). These modules are designed to improve the model's temporal adaptability, robustness, and physical feature integration capabilities.

Within the SPI component, the Adjacent State Connector (ASC) is employed to maintain temporal coherence by capturing sequential dependencies from evolving physical states. The ASC is a lightweight gating mechanism inspired by the Gated Recurrent Unit (GRU) [3], designed to connect adjacent states within our framework. This recurrent structure allows the model to continuously update and track the evolution of physical invariants over time, enabling phase-specific adaptation across different stages of prediction.

The Feature Mixture Module (FMM) integrates a refinement module with a stratified batch sampling strategy to enhance feature robustness. For 1D problems, FMM adopts a lightweight refinement module $\mathcal{D}$ consisting of four convolutional layers with batch normalization and ReLU activation. This setup allows FMM to effectively suppress accumulated noise in the physical invariants while preserving temporal patterns crucial for robust predictions. In more complex 2D scenarios, the FMM leverages a Swin Transformer [4] as a refinement module $\mathcal{D}$ instead. The Swin Transformer applies hierarchical self-attention mechanisms within shifted windows, which facilitates capturing long-range dependencies and multi-scale spatial structures. This makes it particularly suitable for refining noisy SPIs in high-dimensional spatial domains while preserving fine-grained structural features.

The Feature Interaction Module (FIM), integrated into the final SPINO block, enables effective interaction between SPIs and high-level features, thereby enhancing the incorporation of physical priors at deeper layers of the network. It employs an 8-head Multihead Attention mechanism [5], allowing the model to jointly attend to multiple representation subspaces

and reinforce relevant physical patterns during deeper inference stages. To further facilitate information flow and reduce feature dimensionality before attention, two convolutional layers are applied for downsampling, ensuring computational efficiency without sacrificing representational richness.

2.2 Training details

Building upon this architectural framework, we establish an experimental protocol to systematically evaluate SPINO's performance on both 1D and 2D PDE benchmark problems. We use 1000 training instances, 200 validation instances, and 200 test instances. Temporal intervals are divided into 200 frames for training and validation, with input and output frames set to 20 for neural operator and PI encoder. To assess out-of-distribution generalization, test temporal intervals extend to 240 frames, where the last 40 frames appear exclusively in the test set. We define the temporal range within the training set as the "training domain" and the unseen range in the test set as the "future domain".

For optimization, we employ the AdamW optimizer with an initial learning rate of 0.002 for 1D problems and 0.01 for 2D problems, both decayed by a factor of 0.8 every 5,000 epochs over a 20,000 epochs training trajectory. The maximum frequency mode is set to 16 for 1D problems and 12 for 2D problems, aligning with the FNO [2] configuration. All our experiments are implemented in PyTorch [6] on an Ubuntu server with a NVIDIA V100 GPU card. To ensure stability, we repeat each experiment using three random seeds from the set {0, 1, 2} and report the mean value and variance.

2.3 Evaluation metric

The model's performance is evaluated using the average relative L_2 error ($E_{\ell_2}(\%)$) and the relative L_∞ error ($E_{\ell_\infty}(\%)$), both computed over all frames in the training and future domains:

$$E_{\ell_2} = \frac{1}{T}\sum_{t=1}^{T}\frac{\|\tilde{u}_t - u_t\|_2}{\|u_t\|_2}, \quad E_{\ell_\infty} = \frac{\max_{t,x}|\tilde{u}_t(x) - u_t(x)|}{\max_{t,x}|u_t(x)|}. \tag{1}$$

Here, $\tilde{u}_t$ and u_t denote the predicted and ground-truth solutions at time t, with x indicating the spatial location and T the total number of time frames.

3 Details of PI encoder

3.1 Architecture of PI encoder

The PI encoder architecture consists of six layers, including two Fourier layers, two convolutional layers, and two fully connected layers. The Fourier layers extract PDE information in the frequency domain, while the subsequent layers progressively downsample the feature map into a low-dimensional vector. The GeLU activation function is used throughout the network. Notably, the encoder processes only a sub-patch of the PDE field, significantly reducing the required information compared to predicting the full physical field. As a result, the PI encoder serves as a lightweight module for extracting physical invariants (PIs) with fast inference speed.

3.2 Learning strategy for PI encoder

In multi-physical scenarios, high-level physical information θ^i reveals the underlying characteristics of the PDE, enabling clear differentiation among various types. MPNNs [7] show that incorporating the indicator of this physical information can largely improve the generalization capabilities of the neural networks. However, the collection of such physical information can be infeasible or prohibitively expensive in many real-world applications. To address this limitation, PIANO [8] proposes using contrastive learning [9] to learn high-level representations of θ^i from the input raw PDE field $\mathbf{u}^i_{k,t}$, as follows:

$$\mathcal{P} : \mathbb{R}^{n\times t} \to \mathbb{R}^m, \quad \mathcal{P}(\mathbf{u}^i_{k,t}) = \mathbf{e}^i, \tag{2}$$

where $\mathbf{u}^i_{k,t} \in \mathbb{R}^{n\times t}$ denotes the sequence of t consecutive PDE field for the i-th equation instance, n is the number of spatial discretization points, m is the dimension of the encoded representation, $\mathbf{e}^i$ represents the physical invariants, and $\mathcal{P}$ is a parametric network referred to as the PI encoder.

To train the PI encoder, for each PDE sample in training data $\left\{\mathbf{u}_{k,T}^{i}[\Omega]\right\}_{i\in A}$ from $\mathbb{D}_{train}$ with index set A, two groups of cropped patches i.e. $\left\{\mathbf{u}_{k_1,t}^{i}[\Omega_1]\right\}_{i\in A}$ and $\left\{\mathbf{u}_{k_2,t}^{i}[\Omega_2]\right\}_{i\in A}$ are generated. Then, the encoder $\mathcal{P}$ is used to encode the patches to high-dimensional vectors $\mathbf{e}_v^i$ as follows:

$$\mathbf{e}_v^i = \mathcal{P}(\mathbf{u}_{k_v,t}^{i}[\Omega_v]), v \in \{1,2\}\,. \tag{3}$$

In Equation (3), $\mathbf{e}_1^i$ and $\mathbf{e}_2^i$ are derived from the same PDE system governed by θ^i. Thus, mining the mutual information between them can construct the mapping from PDE frames to high-level physical information. Specifically, by considering $\mathbf{e}_1^i$ and $\mathbf{e}_2^i$ as positive samples, and $\mathbf{e}_1^i$ and $\mathbf{e}_2^j$ (where $j \neq i$) as negative samples, the SimCLR loss [9] for training $\mathcal{P}$ can be expressed as follows:

$$\begin{aligned}\mathcal{L}_{\text{SimCLR}} &= -\frac{1}{2|\mathcal{A}|} \\ \sum_{i\in\mathcal{A}} \times &\left[\log \frac{\exp\left(\text{sim}\left(\mathbf{g}(\mathbf{e}_1^i), \mathbf{g}(\mathbf{e}_2^i)\right)/\tau\right)}{\sum_{j\neq i}\sum_{v\in\{1,2\}} \exp\left(\text{sim}\left(\mathbf{g}(\mathbf{e}_1^i), \mathbf{g}(\mathbf{e}_v^j)\right)/\tau\right)}\right. \\ &\left. + \log \frac{\exp\left(\text{sim}\left(\mathbf{g}(\mathbf{e}_1^i), \mathbf{g}(\mathbf{e}_2^i)\right)/\tau\right)}{\sum_{j\neq i}\sum_{v\in\{1,2\}} \exp\left(\text{sim}\left(\mathbf{g}(\mathbf{e}_2^i), \mathbf{g}(\mathbf{e}_v^j)\right)/\tau\right)}\right],\end{aligned} \tag{4}$$

where $\text{sim}(\mathbf{u}, \mathbf{v}) := \mathbf{u}^\top \mathbf{v}/\|\mathbf{u}\|\|\mathbf{v}\|$ denotes the cosine similarity, $\tau > 0$ denotes a temperature parameter, and $\mathbf{g}$ denotes a two-layer multi-layer perceptron (MLP) projection head. The aim of training is to bring the representations governed by the same underlying physical parameters closer in the feature space. After training, the projection head $\mathbf{g}$ is discarded and only $\mathcal{P}$ is used to extract the PI, which is in line with the SimCLR method.

The procedure outlined above is directly applied in our method to train the PI encoder. In contrast to PIANO [8], our approach differs primarily in the way we integrate the pre-trained PI representation into the neural operator, leading to enhanced predictive robustness and accuracy.

3.3 PI encoder training details and efficiency

The PI encoder is pretrained following the contrastive learning paradigm of the PIANO framework [8], with its overall architecture, cropping strategy, and training strategies directly adopted from PIANO. Specifically, we train the encoder for 20,000 epochs using the AdamW optimizer. The initial learning rate is set to 0.001 and decayed by a factor of 0.5 every 4,000 epochs. We adopt a batch size of 512 and a temperature parameter $\tau = 0.5$. This configuration ensures stable convergence while balancing computational efficiency.

To account for the differing characteristics of physical invariants among datasets, we adopt dataset-specific patch configurations, summarized in Supplementary Table 1. For 1D equations (E1–E3), the temporal horizon is set to 200 steps with spatial resolution 64, from which patches of size 32×20 are cropped. For 2D equations (E4–E5), a spatial resolution of 64×64 is used with the same temporal horizon, and larger spatiotemporal patches ($48 \times 48 \times 20$) are adopted to capture richer spatial interactions.

Supplementary Table 1: PI encoder patch specifications for PDE scenarios.

**Scenario**	**Spatial resolution**	**Temporal horizon**	**Patch size (space × time)**	**Notation**
E1	64 grid points	200 steps	32×20	(window = 32, tw_in = 20)
E2	64 grid points	200 steps	32×20	(window = 32, tw_in = 20)
E3	64 grid points	200 steps	32×20	(window = 32, tw_in = 20)
E4	64×64 grid points	200 steps	$48 \times 48 \times 20$	(window = 48, tw_in = 20)
E5	64×64 grid points	200 steps	$48 \times 48 \times 20$	(window = 48, tw_in = 20)

Since the PI types differ across datasets (temporal, boundary, or spatiotemporal invariants), we also employ different cropping strategies tailored to each invariant, as shown in Supplementary Table 2. The categorization of PI types follows the definitions established in PIANO [8], ensuring consistency with prior work. In addition, we report the computational cost of training the PI encoder across datasets in Supplementary Table 3, which shows that the pretraining cost of the PI encoder is negligible compared to the subsequent operator training.

Supplementary Table 2: PI types and corresponding cropping strategies for datasets E1–E5.

**Dataset**	**PI type**	**Spatial cropping**	**Temporal cropping**
E1	Temporal invariant	Same	Different
E2	Temporal invariant	Same	Different
E3	Boundary invariant	Same (at boundary)	Different
E4	Spatiotemporal invariant	Different	Different
E5	Spatiotemporal invariant	Different	Different

Supplementary Table 3: Training cost comparison across datasets E1–E5. The table reports the average training time per epoch (in seconds), the model size (in millions of parameters), and the total number of training epochs.

Dataset	Training Time (s)	Model Size (M)	Epoch
E1	0.074	0.051	20000
E2	0.081	0.051	20000
E3	0.076	0.051	20000
E4	0.188	0.231	20000
E5	0.183	0.231	20000

3.4 Ablation of PI encoder

To evaluate the validity and rationale of using patches as inputs to PI encoder, we conduct an ablation study on patch size and cropping strategy, with results summarized in Supplementary Table 4 using the 1D Burgers' equation (E1) as an illustrative case. In our framework, the spatial dimension of the Burgers' equation is 64, which we partition into two equal patches of size 32, each covering half of the domain. This choice naturally yields two non-overlapping patches of size 32 × 20 in space-time, which is adopted as the default configuration in 1D case in our experiments.

We further tested alternative patch sizes, including 16 × 20 and 48 × 20. The smaller size

Supplementary Table 4: Ablation on patch size and cropping strategy for the PI encoder (Burgers' equation).

**Patch size**	**Sampling strategy**	**Training domain**		**Future domain**	
		$E_{\ell_2}(\%)$	$E_{\ell_\infty}(\%)$	$E_{\ell_2}(\%)$	$E_{\ell_\infty}(\%)$
16 × 20	Space: Same, Time: Different	0.668	1.021	0.845	1.312
32 × 20 (ours)	Space: Same, Time: Different	**0.227**	**0.361**	**0.244**	**0.403**
48 × 20	Space: Same, Time: Different	0.282	0.440	0.329	0.517
32 × 20	Space: Different, Time: Same	0.560	0.785	0.660	0.955
32 × 20	Space: Different, Time: Different	0.703	0.968	0.907	1.258

results in insufficient spatial coverage and higher variance, leading to degraded accuracy, while the larger size for 1D case introduces redundancy. As shown in Supplementary Table 4, the 32×20 configuration consistently yields the lowest errors across both the training and future domains.

In addition to patch size, we examined different cropping strategies along spatial and temporal dimensions. Since the physical invariants in Burgers' equation are primarily temporal invariants, the most effective strategy is to crop from the same spatial domain but from different temporal windows of the same instance. In contrast, strategies involving different spatial domains, either at the same or different times, lead to a significant increase in prediction error, as they fail to satisfy the governing equation and reduce PI encoder's ability to learn stable physical-invariant features.

4 More ablation on key modules

In the main text, we have presented ablation studies to verify the effectiveness of the proposed architecture. Before analyzing the impact of individual modules, we first examine the trade-off between model complexity and predictive accuracy. Building on this, we then conduct further ablation studies on the three key modules—Sliding Physical Invariants (SPI), Feature Mixture Module (FMM), and Feature Interaction Module (FIM). These extended studies provide deeper insights into the robustness and necessity of each design choice.

4.1 Trade-off between model complexity and predictive accuracy

While SPINO achieves noticeable improvements in predictive accuracy, these gains are accompanied by increased computational cost due to the enlarged model size. As reported in the main text, the parameter count reaches 1.118 million for 1D problems and 11.873 million for 2D problems. This increased complexity results in training and inference times that are respectively 2-7× and 2-15× longer than those of state-of-the-art methods, while inference remains within seconds. This observation raises the practical question of whether the accuracy gains justify the additional computational overhead, and how the trade-off between model complexity and

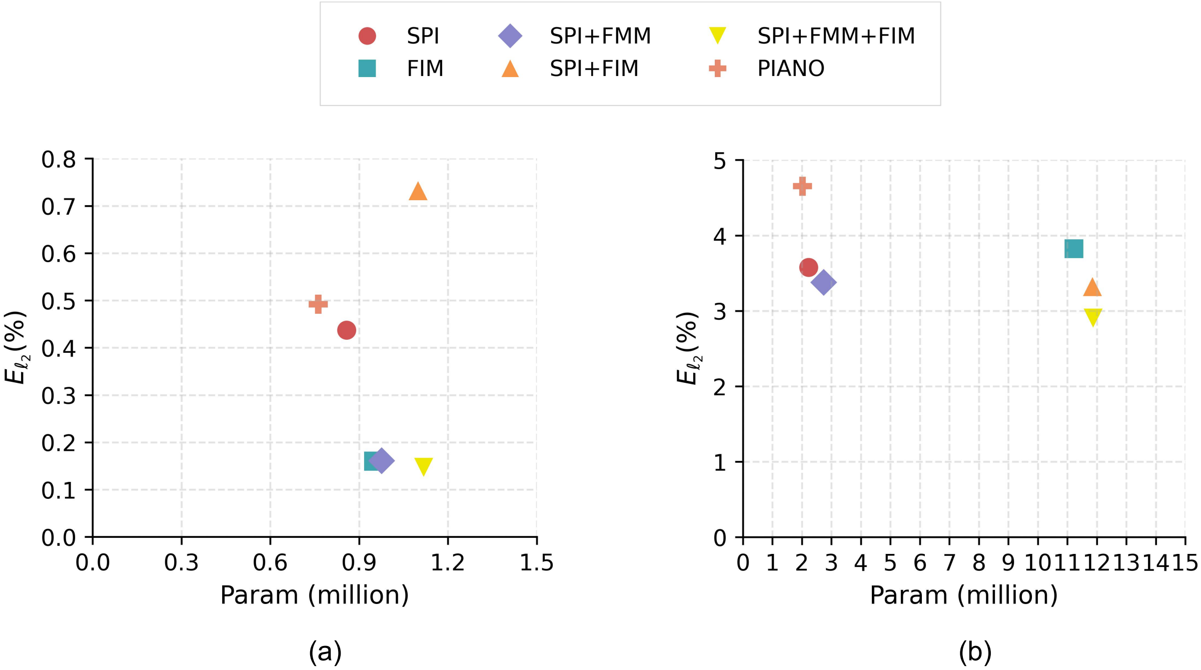

Supplementary Figure 2: Trade-off between model complexity (in terms of number of parameters) and prediction error. (a) 1D CDE. (b) 2D NSE. Each marker represents a distinct model variant, including SPI, FIM, SPI+FMM, SPI+FIM, and SPI+FMM+FIM, together with the baseline PIANO model for comparison.

prediction accuracy evolves when different modules of SPINO are incrementally introduced.

To answer this question, we conduct a comprehensive module-wise ablation study that progressively incorporates SPI, FMM, and FIM into the architecture. Each variant is evaluated against the PIANO baseline under comparable parameters, with experiments carried out on two representative benchmarks: the 1D Convection-Diffusion Equation (CDE) and the 2D Navier-Stokes Equation (NSE). The results, summarized in Supplementary Figure 2. On the 1D CDE benchmark, specifically, the simplest SPINO (SPI only) achieves an 11.7% improvement in accuracy with a 0.12× increase in parameter count. The lightweight SPINO variant (SPI+FMM) attains a favorable balance between efficiency and accuracy, yielding a 67.2% increase in prediction accuracy with a 0.28× increase in parameter count. Further integrating all components, the full SPINO (SPI+FMM+FIM) further achieves the lowest error, offering a 70.1% accuracy improvement at the cost of a 0.46× increase in parameter count.

On the more challenging 2D NSE benchmark, the different SPINO variants exhibit clear

differences in balancing accuracy and model complexity. Specifically, the simplest SPINO (SPI only) achieves a 23.1% improvement in accuracy with a 0.23× increase in parameter count. The lightweight SPINO variant (SPI+FMM) attains a favorable balance between efficiency and accuracy, yielding a 27.4% increase in prediction accuracy with a 0.35× increase in parameter count. Finally, the full SPINO (SPI+FMM+FIM) achieves the lowest error among all configurations, yielding a 37.5% improvement in prediction accuracy. This gain, however, comes at the cost of a 4.87× increase in parameter count due to the inclusion of the FIM module, which expands the input feature dimensionality.

These findings suggest that SPINO variants not only improve forecasting fidelity but also offer flexible accuracy–efficiency choices depending on resource availability. For computationally constrained scenarios, the lighter SPINO variants preserve most of the performance advantages while lowering the parameter count and runtime. Conversely, in applications where long-term accuracy and stability are critical, the full SPINO model provides substantial benefits that outweigh the additional computational burden.

4.2 Ablation on Sliding Physical Invariants (SPI)

To further demonstrate the effectiveness of SPI, we utilize the pretrained PI encoder to generate the corresponding attention weights from SPI. We describe experiments to investigate SPI on the E3 (CDE) and E4 (NSE) data; specifically, whether SPI can capture the time-varying physical characteristics within the PDE system. As shown in Supplementary Figure 3(c) and (d), the physical invariant representation continuously evolves over time. Moreover, as the PDE system becomes more complex, such as a high-dimensional problem, SPI exhibits even greater variations over time. Specifically, as time T increases, the SPI gradually deviates from its initial state, sometimes becoming entirely distinct from the initial PI, as illustrated in Supplementary Figure 3(d). This significant shift suggests that the initial PI is no longer sufficient to guide the learning process of the neural operator at later time steps, as it fails to capture the evolving physical properties of the system.

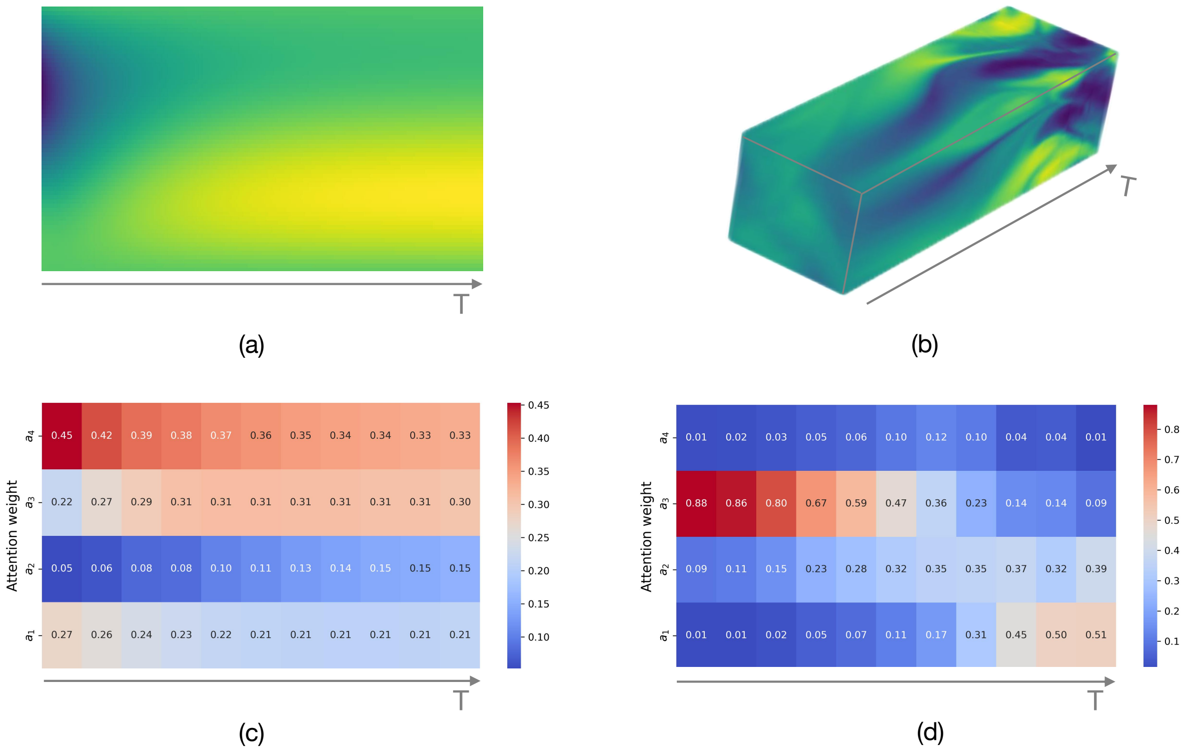

Supplementary Figure 3: Predicted solutions and attention weight visualizations for CDE and NSE. (a) Predicted solution of the CDE. (b) Predicted solution of the NSE. (c) Evolution of attention weights in the CDE. (d) Evolution of attention weights in the NSE.

4.3 Ablation on Feature Mixture Module (FMM)

Before integrating the Feature Mixture Module (FMM), we hypothesized that enhancing the robustness of PI encoder $\mathcal{P}$ would improve the quality of PI generation. Additionally, strengthening the neural operator's capability to process noisy input data was expected to further enhance the overall performance. To explore these possibilities, we conducted experiments with two approaches: (1) adding noise to the PIs during the pretraining phase, and (2) introducing noise to the input data $\mathbf{u}$ during the training phase.

Supplementary Table 5 presents a detailed comparison of the $E_{\ell_2}(\%)$ error under varying noise levels ($\sigma_1 = 0.1$, $\sigma_2 = 0.01$, $\sigma_3 = 0.001$) on the E3 (CDE) and E4 (NSE) datasets. The upper part of the table shows three ablation configurations with different combinations of pretraining and training. When only pretraining is used (first row), moderate performance is

Supplementary Table 5: $E_{\ell_2}(\%)$ comparison under varying noise levels ($\sigma_1 = 0.1$, $\sigma_2 = 0.01$, $\sigma_3 = 0.001$) on the E3 (CDE) and E4 (NSE) datasets. ✔ denotes that noise is introduced during the pretraining or training stage. "SPINO without FMM" denotes our model without the Feature Mixture Module, while "SPINO (ours)" refers to the full version. Lower values indicate better refinement performance. The best results in each task are highlighted in **bold**.

pretrain	train	E3 (CDE)			E4 (NSE)		
		σ_1	σ_2	σ_3	σ_1	σ_2	σ_3
✔	–	0.513	0.413	0.399	3.865	3.460	3.317
–	✔	0.182	0.147	0.195	19.883	3.574	3.482
✔	✔	0.333	0.185	0.248	5.319	3.395	3.436
SPINO without FMM		0.732			3.321		
SPINO (ours)		**0.147**			**2.904**		

observed on both datasets. It can be observed that when only training is enabled (second row), the model achieves relatively low error on E3 but performs poorly on E4 under high noise (σ_1), indicating limited generalization. Combining both pretraining and training (third row) improves stability across datasets, particularly under moderate noise conditions. The bottom part of the table compares two variants of the proposed SPINO model. "SPINO without FMM" performs better than the ablation baselines, confirming the strength of the SPINO backbone. However, "SPINO (ours)"–which incorporates the Feature Mixture Module (FMM)–consistently achieves superior performance across all noise levels and datasets, with error reductions from 0.732% to 0.147% on E3 and from 3.321% to 2.904% on E4, respectively. This demonstrates the effectiveness of FMM in enhancing the model's robustness to noise and improving refinement quality under complex physical dynamics.

To further evaluate the robustness of the proposed method, we consider a more challenging setting where Gaussian noise with varying intensities is injected at the batch level. Specifically, the standard deviation σ of the Gaussian noise is randomly sampled from the candidate set $\{10^{-3}, 3 \times 10^{-3}, 10^{-2}, 3 \times 10^{-2}, 10^{-1}\}$ according to a uniform probability distribution. In this way, each sample in a batch may be perturbed by noise of different intensities. This strategy simulates more diverse and uncertain noise conditions, providing a comprehensive assessment of the model's refinement capability and robustness. As reported in Supplementary Table 6, the full SPINO model with FMM again achieves the lowest errors across datasets, consistent

with the fixed-noise experiments. More importantly, the advantage of FMM becomes even more pronounced under mixed noise, providing stronger evidence that FMM is crucial for ensuring robustness when dealing with diverse and uncertain noise intensities.

Supplementary Table 6: $E_{\ell_2}(\%)$ results under mixed noise conditions (simultaneous injection of $\{10^{-3}, 3\times10^{-3}, 10^{-2}, 3\times10^{-2}, 10^{-1}\}$) for the E3 (CDE) and E4 (NSE) datasets. "✔" indicates that mixed noise is introduced during the pretraining or training stage. "SPINO without FMM" denotes the model variant without the Feature Mixture Module, while "SPINO (ours)" refers to the full version. Lower values indicate better refinement performance. The best results in each task are highlighted in **bold**.

Model	pretrain	train	E3 (CDE)	E4 (NSE)
Mixed noise	✔	–	0.658	3.717
	–	✔	0.172	4.176
	✔	✔	0.228	3.410
SPINO without FMM	–	–	0.732	3.321
SPINO (ours)	–	–	**0.147**	**2.904**

4.4 Ablation on Feature Interaction Module (FIM)

We further ablate the Feature Interaction Module by comparing cross-attention (CA) and self-attention (SA), each with or without downsampling (DS) (Supplementary Table 7). In the training domain, SA combined with DS consistently achieves the lowest errors on both benchmarks (E3 and E4), indicating its effectiveness in enhancing feature integration and spatial–temporal alignment. In the future domain, the performance trends diverge: on the simpler one-dimensional E3, CA without DS attains the best accuracy, suggesting that DS may over-smooth low-frequency signals and weaken phase tracking under autoregressive drift. In contrast, on the more complex E4, SA with DS remains superior, demonstrating its advantage in handling high-dimensional dynamics and mitigating distribution shift. These results highlight the complementary roles of attention type and downsampling, and validate the design of FIM for robust long-term prediction.

Supplementary Table 7: Ablation studies on the Feature Interaction Module. "CA" represents cross-attention module. "SA" represents self-attention module. "DS" represents downsampling. The best results in each tasks are highlight in **bold**.

Method	training domain				future domain			
	E3 (CDE)		E4 (NSE)		E3 (CDE)		E4 (NSE)	
	$E_{\ell_2}(\%)$	$E_{\ell_\infty}(\%)$	$E_{\ell_2}(\%)$	$E_{\ell_\infty}(\%)$	$E_{\ell_2}(\%)$	$E_{\ell_\infty}(\%)$	$E_{\ell_2}(\%)$	$E_{\ell_\infty}(\%)$
CA w/o DS	0.185	0.392	3.424	6.883	**0.275**	**0.551**	21.943	42.943
CA w/ DS	0.282	0.527	2.926	5.855	0.590	0.955	16.827	37.023
SA w/o DS	0.203	0.433	3.418	6.848	0.311	0.634	20.386	47.024
SA w/ DS	**0.147**	**0.338**	**2.904**	**5.797**	0.377	0.555	**16.048**	**36.508**

5 Quantitative analysis of error accumulation

In long-term auto-regressive prediction, even small deviations at each rollout step can gradually accumulate and amplify, eventually dominating the prediction and leading to unstable solutions. To quantitatively assess this phenomenon, we evaluate the point-by-point prediction error over 220 evolving steps on Burgers' equation.

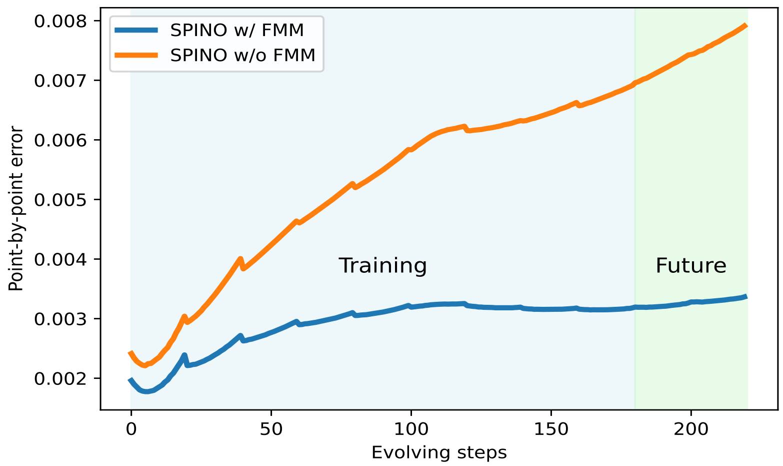

Supplementary Figure 4: Point-by-point error of Burgers' equation from time step 0 to 219. Note that t = 0 to 179 corresponds to training domain, while t = 180 to 219 represents future domain. All errors are measured in terms of the L_2 norm.

As shown in Supplementary Figure 4, the model without FMM exhibits a near-linear increase in error during the training domain (t = 0–179) and a pronounced acceleration after

entering the future domain (t = 180–219), with the error rising by approximately 0.8×10^{-2}. This behavior indicates that once the model steps beyond the supervised region, accumulated deviations are no longer corrected and rapidly escalate, severely impairing prediction stability. In contrast, the model equipped with FMM exhibits a substantially flatter error trajectory, with the increase in the future domain restricted to approximately $0.3 \sim 0.4 \times 10^{-2}$, resulting in an average growth rate nearly two times lower. This comparison provides clear evidence that error accumulation is the key limiting factor for reliable extrapolation, and demonstrates that mechanisms like FMM are essential to effectively suppress error propagation and stabilize long-horizon rollouts. Beyond numerical improvement, this analysis highlights the broader contribution of our framework: By directly addressing the challenge of error accumulation, SPINO establishes a more robust foundation for scientific forecasting in complex dynamical systems.

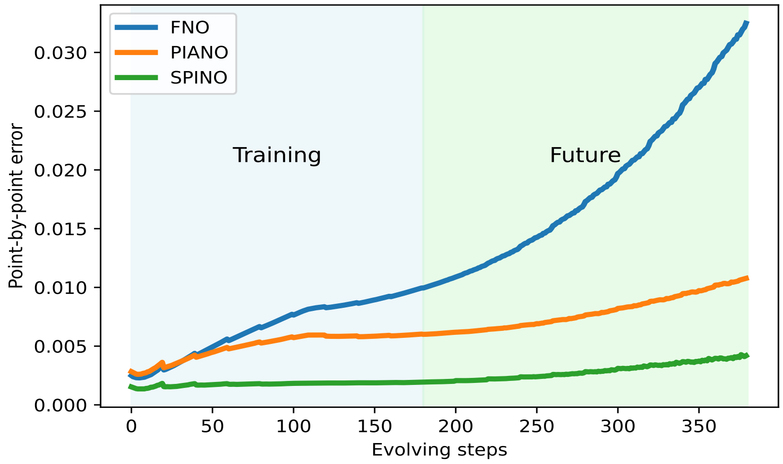

Supplementary Figure 5: Point-by-point error of Burgers' equation from time step 0 to 379, where t = 0 to 179 corresponds to the training domain and t = 180 to 379 represents the future domain. Point-by-point error curves of FNO, PIANO, and SPINO are reported over both training and extrapolation horizons. All errors are measured in terms of the L_2 norm.

To further address the concern of long-term prediction capability, we extend the extrapolation horizon to twice the training final time (2×), significantly longer than the originally reported 20% extension. Specifically, for Burgers' equation, the training horizon covers $t = 0$ to 179, and we evaluate the models up to $t = 379$, which corresponds to an extrapolation length equal to the entire training horizon. Supplementary Figure 5 presents the point-by-point error

curves of FNO, PIANO, and SPINO over both the training and extrapolation horizons. The results show that FNO suffers from rapid error growth, with its point-by-point error increasing from approximately 0.002 at $t = 0$ to over 0.030 by the end of the extrapolation horizon, representing more than a 15-fold increase. PIANO exhibits slower growth, starting at about 0.0025 and reaching around 0.010 at $t = 379$, corresponding to roughly a 4-fold increase. In contrast, SPINO maintains substantially lower errors, rising only from 0.0015 to about 0.005 across the entire horizon, a modest 3-fold increase, thereby demonstrating remarkable stability and robustness in long-term prediction compared with the baseline methods. This indicates that SPINO achieves stable predictions even when extrapolated far beyond the training horizon, thereby substantiating our claim of long-term predictive capability.

6 Extrapolation with progressive data availability

Motivated by real-world applications that demand continuous updates from sequential observations, such as weather forecasting and aerodynamic control systems, we systematically assess SPINO's predictive performance as the available time gradually extends. The results are presented in Supplementary Table 8. We find that the predictive performance of SPINO for future domains continues to improve with the increasing amount of input data available in the testing phase, and the prediction accuracy is always better than PIANO [8]. Specifically, for E1 (Burgers), SPINO reduced the error from 0.286% to 0.038% as the amount of available input data increased, while PIANO reduced the error from 0.492% to 0.072%. For E4 (NSE), the error

Supplementary Table 8: Extrapolation results with respect to available data from different time intervals (0-20, 0-40, 0-60, ...) for E1 (Burgers) and E4 (NSE) datasets. The best results in each task are highlighted in bold.

		$E_{\ell_2}(\%)$ with available data from varying numbers of frames									
Data	Method	20	40	60	80	100	120	140	160	180	200
E1	PIANO	0.492	0.411	0.359	0.315	0.273	0.232	0.191	0.151	0.111	0.072
	SPINO(ours)	**0.286** (↓ 41.8%)	**0.186** (↓ 54.7%)	**0.140** (↓ 61.0%)	**0.124** (↓ 60.6%)	**0.102** (↓ 62.6%)	**0.093** (↓ 59.9%)	**0.073** (↓ 61.7%)	**0.064** (↓ 57.6%)	**0.050** (↓ 54.9%)	**0.038** (↓ 47.2%)
E4	PIANO	17.393	17.241	16.434	16.057	15.205	14.745	13.883	13.340	12.464	11.577
	SPINO(ours)	**16.048** (↓ 7.7%)	**13.110** (↓ 23.9%)	**11.929** (↓ 27.4%)	**11.332** (↓ 29.4%)	**10.973** (↓ 27.8%)	**10.833** (↓ 26.5%)	**10.674** (↓ 23.1%)	**10.557** (↓ 20.8%)	**10.284** (↓ 17.4%)	**9.727** (↓ 15.9%)

in the future domain decreased as the amount of available input data increased, from 16.048% to 9.727%, while PIANO reduced the error from 17.393% to 11.577%. The aforementioned experiment highlights SPINO's advantage in scenarios where future data becomes progressively available, enabling the model to refine its predictions and enhance its performance over time.

7 Super-resolution capability of SPINO

All models presented in the main text were trained under a fixed spatial resolution of 64 (for 1D PDEs) or 64×64 (for 2D PDEs). However, in practical applications, the available data may not always be obtained at the same ideal resolution; instead, it may be coarser or finer. We refer to the ability of a model to generalize across varying input resolutions as its *super-resolution capability*. In this section, we use the CDE as a representative example to evaluate the performance of SPINO under different input sizes and compare it with FNO. All models are trained only at the resolution of 64. Note that SPINO here denotes a variant of our method, where FMM and FIM modules are excluded.

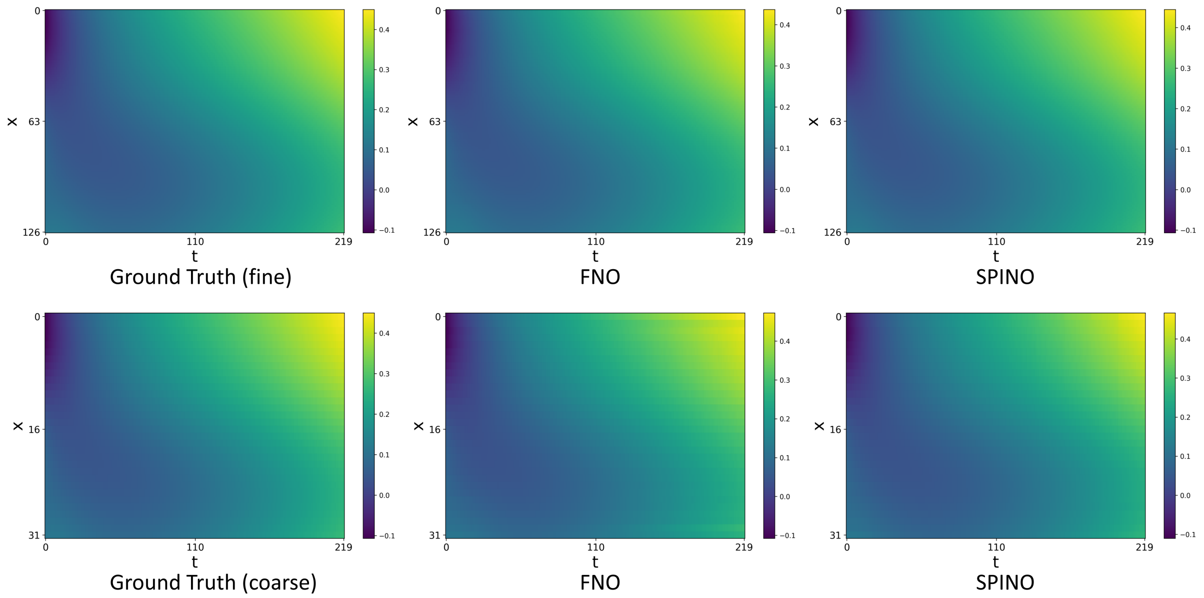

Supplementary Figure 6: Visualization of predicted solutions by FNO and SPINO under varying resolutions. Both models are trained only at a fixed resolution of 64, and tested at finer (128) and coarser (32) resolutions.

Supplementary Table 9: Quantitative comparison of prediction accuracy between FNO and SPINO under varying spatial resolutions for the CDE. All models are trained at resolution 64. The up-sampling strategy indicates resampling inputs to the training resolution (64), applying the model, and then upsampling predictions back to the target resolution.

Method	$E_{\ell_2}(\%)$ in training domain			$E_{\ell_2}(\%)$ in future domain		
	R=32	R=64	R=128	R=32	R=64	R=128
FNO	7.55	1.27	3.18	19.73	1.47	5.63
SPINO	6.08	0.42	2.57	16.54	0.65	5.57
FNO w/ up-sampling	1.24	1.27	1.26	1.43	1.47	1.47
SPINO w/ up-sampling	**0.41**	**0.42**	**0.42**	**0.64**	**0.65**	**0.65**

As illustrated in Supplementary Figure 6, it can be observed that both FNO and our method SPINO can be applied to data with finer (128) or coarser (32) resolutions, and the predicted solutions broadly capture the correct trend and overall shape of the ground truth. However, when examining the quantitative accuracy, as summarized in the first two rows of Supplementary Table 9, it becomes evident that input resolution has a substantial impact on prediction errors. This phenomenon primarily results from the structural design of the Fourier layer in both FNO and SPINO, where the learned linear transformation is constrained to a fixed number of frequency modes. As a result, when the input resolution differs from the training setting, the correspondence between frequency indices and physical frequencies shifts, causing the learned weights to act on different frequency bands. This resolution–frequency mismatch becomes particularly pronounced when high-frequency components dominate the PDE dynamics, as such modes are more sensitive to resolution changes and prone to rapid error accumulation.

To further support this interpretation, we conducted an additional set of experiments, as reported in the last two rows of Supplementary Table 9. Specifically, when input data with non-64 resolutions were first resampled to the training resolution (64), processed by the model, and then resampled back to their original resolutions, the resulting accuracy was nearly identical to that obtained directly at the training resolution. This finding confirms that the observed predictive degradation primarily stems from a resolution-frequency mismatch in the operator layer, rather than from any intrinsic limitation of SPINO in handling multi-scale data. These results

further emphasize that, when frequency alignment is properly addressed, SPINO demonstrates strong and consistent super-resolution capability, particularly under coarse-to-fine evaluation scenarios.

8 Theoretical analysis of SPINO: error propagation and stability in long-term predictions

In this section, we present a theoretical analysis of the long-term prediction stability of SPINO for time-dependent systems. We begin by fixing the notations: let $S \subset \mathbb{R}^D$ denote the state space, where D represents the effective dimension of the discretized system. Specifically, for a one-dimensional spatial domain discretized into N_x grid points with a temporal input window of length N_t, the dimension is given by

$$D = N_x \times N_t. \tag{5}$$

For instance, when $N_x = 64$ and $N_t = 20$, we obtain $D = 1280$, which corresponds to the concatenation of 20 consecutive frames, each of size 64. Time is treated as discrete and indexed by $k \in \mathbb{N}$.

Let the ground-truth one-step evolution be $\mathcal{F} : S \to S$ and the learned one-step predictor of SPINO be $\mathcal{G} : S \to S$. The true and predicted trajectories are generated by

$$x^\star_{k+1} = \mathcal{F}(x^\star_k), \qquad \tilde{x}_{k+1} = \mathcal{G}(\tilde{x}_k), \tag{6}$$

with initial condition $x^\star_0 \in S$ and $\tilde{x}_0 \in S$, and for $k = 0$, $x^\star_0 = \tilde{x}_0$. Starting from any initial state $\tilde{x}_0 \in S$, the model rollouts produce:

$$x^\star_0 = \tilde{x}_0 \to \tilde{x}_1 \to \tilde{x}_2 \to \tilde{x}_3 \to \cdots, \tag{7}$$

Then, the prediction error at the k-th step is defined as

$$e_k := \frac{\|\tilde{x}_k - x_k^\star\|}{\|x_k^\star\|}, \tag{8}$$

where $\|\cdot\|$ denotes the L_2 norm on $\mathbb{R}^D$. Before proceeding with the analysis, we state the following assumption.

Assumption 8.1. *There exists a compact forward-invariant set $M \subset S$ such that, for any initial state*

$$x_0^\star \in M \quad \textit{and} \quad \tilde{x}_0 \in M,$$

we have

$$x_k^\star \in M,\ \tilde{x}_k \in M, \quad \forall k \geq 0,$$

and all regularity constants (e.g., Lipschitz constants, local approximation bounds) are taken over M.

This assumption guarantees that both the true and predicted trajectories remain confined within a bounded region of the state space. Consequently, all Lipschitz and regularity constants can be chosen uniformly over M, preventing uncontrolled growth of constants and ensuring that the subsequent error estimates remain valid along the entire trajectory.

By adding and subtracting $\mathcal{G}(x_k^\star)$ and applying the triangle inequality,

$$\begin{aligned} e_{k+1} &= \frac{\|\mathcal{G}(\tilde{x}_k) - \mathcal{F}(x_k^\star)\|}{\|x_{k+1}^\star\|} \\ &= \frac{\|\left(\mathcal{G}(\tilde{x}_k) - \mathcal{G}(x_k^\star)\right) + \left(\mathcal{G}(x_k^\star) - \mathcal{F}(x_k^\star)\right)\|}{\|x_{k+1}^\star\|} \\ &\leq \frac{\|\mathcal{G}(\tilde{x}_k) - \mathcal{G}(x_k^\star)\| + \|\mathcal{G}(x_k^\star) - \mathcal{F}(x_k^\star)\|}{\|x_{k+1}^\star\|} \\ &= \underbrace{\frac{\|\mathcal{G}(x_k^\star) - \mathcal{F}(x_k^\star)\|}{\|x_{k+1}^\star\|}}_{\alpha_k} + \frac{\|\mathcal{G}(\tilde{x}_k) - \mathcal{G}(x_k^\star)\|}{\|x_{k+1}^\star\|}. \end{aligned} \tag{9}$$

Here α_k is the one-step approximation error at the true state, normalized by $\|x_{k+1}^\star\|$.

On the compact set M, let L_k denote the local Lipschitz constant of $\mathcal{G}$, such that:

$$\|\mathcal{G}(x) - \mathcal{G}(y)\| \leq L_k \|x - y\|, \quad \forall x, y \in M. \tag{10}$$

Therefore, by applying the Lipschitz continuity of $\mathcal{G}$, we obtain:

$$\frac{\|\mathcal{G}(\tilde{x}_k) - \mathcal{G}(x_k^\star)\|}{\|x_{k+1}^\star\|} \leq \frac{L_k \|\tilde{x}_k - x_k^\star\|}{\|x_{k+1}^\star\|}. \tag{11}$$

In practice, however, the error amplification incurred in a particular rollout step is typically smaller than the worst-case constant L_k. We therefore introduce an effective amplification factor

$$\beta_k := \frac{\|\mathcal{G}(\tilde{x}_k) - \mathcal{G}(x_k^\star)\|}{\|\tilde{x}_k - x_k^\star\|} \cdot \frac{\|x_k^\star\|}{\|x_{k+1}^\star\|}, \qquad \text{whenever } \|\tilde{x}_k - x_k^\star\| \neq 0, \tag{12}$$

and set $\beta_k := 0$ when $\tilde{x}_k = x_k^\star$.

By construction, $\beta_k \leq L_k \frac{\|x_k^\star\|}{\|x_{k+1}^\star\|}$, and it measures the actual (normalized) sensitivity of the predictor at the current step.

Using this definition in Equation (9) and noting that

$$\frac{\|\mathcal{G}(\tilde{x}_k) - \mathcal{G}(x_k^\star)\|}{\|x_{k+1}^\star\|} = \beta_k \frac{\|\tilde{x}_k - x_k^\star\|}{\|x_k^\star\|} = \beta_k e_k, \tag{13}$$

we obtain the fundamental recursive error inequality in relative form:

$$e_{k+1} \leq \alpha_k + \beta_k e_k. \tag{14}$$

Here, α_k quantifies the one-step bias of the learned operator, while β_k captures the step-wise error amplification along the trajectory. This refinement not only provides a more accurate description of error dynamics but also enables empirical estimation of α_k and β_k from rollout experiments.

Iterating the one–step inequality yields the standard variation–of–constants expansion:

$$e_k \le \sum_{j=0}^{k-1} \alpha_j \Big(\prod_{i=j+1}^{k-1} \beta_i \Big) \quad (k \ge 1), \tag{15}$$

with the convention that empty products equal 1.

Sliding Physical Invariants (SPIs) Guided Dynamic Convolution. Physical Invariants (PIs) are extracted from the input PDE sequence by PI encoder $\mathcal{P}$, which produces high-level representations:

$$\mathcal{P} : \mathbb{R}^D \to \mathbb{R}^m, \quad \mathcal{P}(\tilde{x}_k) = \mathbf{e}^k. \tag{16}$$

These invariants encode essential equation-related information and are used to generate the parameters for the dynamic convolution, thereby guiding the processing of the input. In the PIANO framework, the prediction step is formulated as

$$\tilde{x}_{k+1} = \mathcal{G}_\theta(\tilde{x}_k), \qquad \theta \sim p(\theta \mid \mathbf{e}^0), \tag{17}$$

where the dynamic convolution parameters θ of the operator $\mathcal{G}$ are statically determined by the invariant representation $\mathbf{e}^0$. However, this design suffers from a key limitation in autoregressive prediction: as k increases, the one-step error α_k grows progressively uncontrolled. Since $\mathbf{e}^0$ is extracted solely from the initial state $\tilde{x}_0$, the discrepancy between $\tilde{x}_0$ and $\tilde{x}_k$ gradually enlarges during system evolution, especially in future domains.

To address this issue, we propose the *Sliding Physical Invariants (SPI)*, where the prediction step is reformulated as

$$\tilde{x}_{k+1} = \mathcal{G}_\theta(\tilde{x}_k), \qquad \theta \sim p(\theta \mid \mathbf{e}^k, \mathbf{e}^{k-1}). \tag{18}$$

SPI is aligned with the autoregressive nature of the task: it generates the dynamic convolution parameters from the most recent predictions, ensuring that the parameterization remains consistent with the current state. Compared with PIANO, SPI is expected to reduce the one-step

bias. Under the mild assumption that

$$\alpha_k^{SPI} \leq \alpha_k^{PI}, \qquad \beta_k^{SPI} = \beta_k^{PI}, \tag{19}$$

the error recursion $e_{k+1} \leq \alpha_k + \beta_k e_k$ immediately implies

$$e_k^{SPI} \leq e_k^{PI}, \qquad \forall k, \tag{20}$$

indicating that SPI achieves more stable and accurate long-term predictions.

To further validate the above assumption empirically, we compute the one-step bias α_k and amplification factor β_k for both SPI and PI across different prediction steps k on the Burgers' equation with varying external forces f (experiment with random seed 0, which is consistently used in the following experiments). The comparative results are illustrated in Supplementary Figure 7. The results confirm the theoretical assumption: the one-step error coefficient α_k of SPI remains consistently smaller than that of PI, while the amplification factor β_k exhibits negligible difference between the two. Also, we illustrate the step-wise prediction error e_k in Supplementary Figure 8, which further validates the assumption empirically.

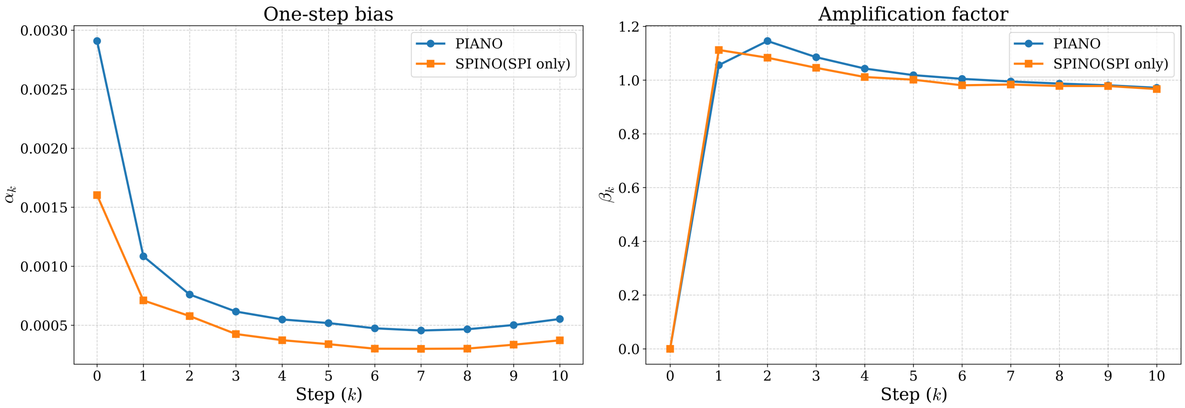

Supplementary Figure 7: Comparison of the one-step error coefficient α_k and the amplification factor β_k between the proposed Sliding Physical Invariants (SPI) and the original Physical Invariant (PI) schemes on the Burgers' equation with varying external forces f.

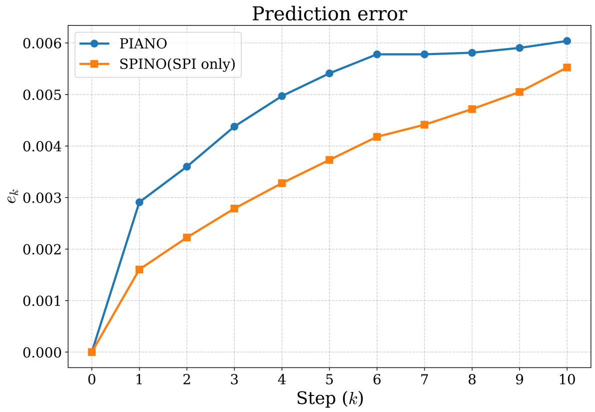

Supplementary Figure 8: Comparison of the step-wise prediction error e_k between the proposed Sliding Physical Invariants (SPI) and the original Physical Invariant (PI) schemes on the Burgers' equation with varying external forces f.

Feature Mixture Module (FMM). Recall that the recursive error inequality involves both the one-step bias α_k and the amplification factor β_k. The Feature Mixture Module (FMM) is designed to improve long-term stability by reducing β_k. Specifically, FMM attempts to reduce β_k from two complementary aspects: (i) enhancing the quality of SPIs, and (ii) increasing the robustness of the operator $\mathcal{G}$ against noisy or imperfect invariants. Without such mechanisms, noisy predictions may propagate through PI encoder $\mathcal{P}$, producing inferior SPIs and consequently increasing β_k, thereby accelerating error accumulation.

Formally, let the noisy prediction at step k be $\tilde{x}_k$. Since $\mathcal{P}$ is pre-trained only on exact solutions, it is sensitive to the distribution shift between $\tilde{x}_k$ and $x_k^\star$. To alleviate this, FMM employs a refinement module $\mathcal{D}$ before feeding the prediction into $\mathcal{P}$:

$$\tilde{x}_{k+1} = \mathcal{G}_\theta(\tilde{x}_k), \qquad \theta \sim p\big(\theta \mid \mathcal{P}(\mathcal{D}(\tilde{x}_k)),\ \mathcal{P}(\mathcal{D}(\tilde{x}_{k-1}))\big). \tag{21}$$

This design ensures that $\mathcal{P}$ receives refined fields $\mathcal{D}(\tilde{x}_k)$, thereby improving the stability of SPI extraction.

In addition, FMM improves the robustness of $\mathcal{G}$ against imperfect invariants by mixing exact

and noisy SPIs during training. This is realized via stratified batch sampling:

$$\begin{cases} x_k^\star \underset{\mathbf{e}^k}{\longmapsto} \tilde{x}_{k+1} \underset{\tilde{\mathbf{e}}^{k+1}}{\longmapsto} \tilde{x}_{k+2}, \\ x_k^\star \underset{\mathbf{e}^k}{\longmapsto} \tilde{x}_{k+1} \underset{\mathbf{e}^{k+1}}{\longmapsto} \tilde{x}_{k+2}, \end{cases} \tag{22}$$

so that $\mathcal{G}$ is trained to handle both clean and perturbed invariants. This reduces its effective sensitivity to noise, thereby lowering the amplification factor β_k.

In summary, FMM contributes to long-term stability through a dual effect: (i) refining-based enhancement of SPI quality reduces the influence of noisy input, while (ii) mixed-invariant training improves the robustness of $\mathcal{G}$ against imperfect invariants. Together, these mechanisms reduce error accumulation and enhance the robustness of the predictor. To further evaluate the effectiveness of FMM, we compute both α_k and β_k across different steps k with and without the module on the Burgers' equation with varying external forces f. The comparative results are illustrated in Supplementary Figure 9. The results demonstrate that FMM consistently reduces the amplification factor β_k, with a minor increase observed in α_k. The final step-wise prediction error is shown in Supplementary Figure 10. The results indicates that FMM effectively mitigates error propagation and enhances the long-term stability of the predictor.

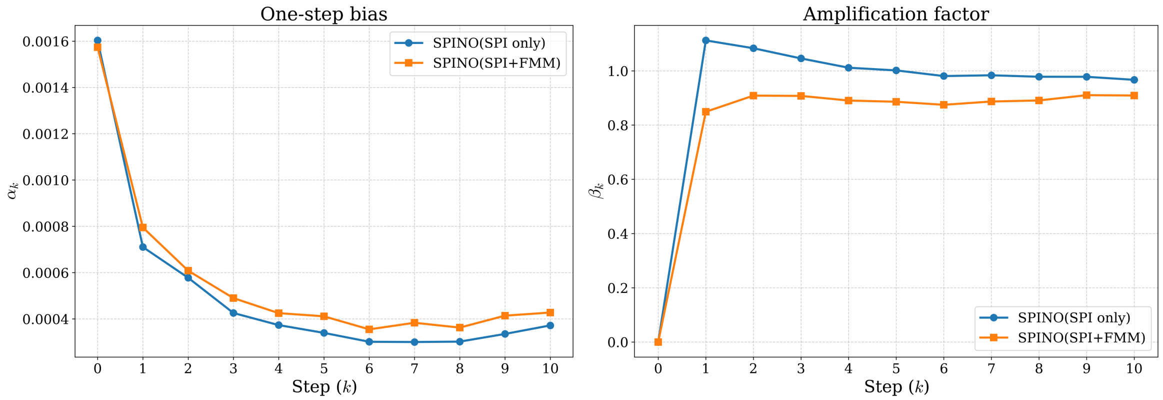

Supplementary Figure 9: Comparison of the one-step error coefficient α_k and amplification factor β_k between the models with and without the Feature Mixture Module (FMM) on the Burgers' equation with varying external forces f.

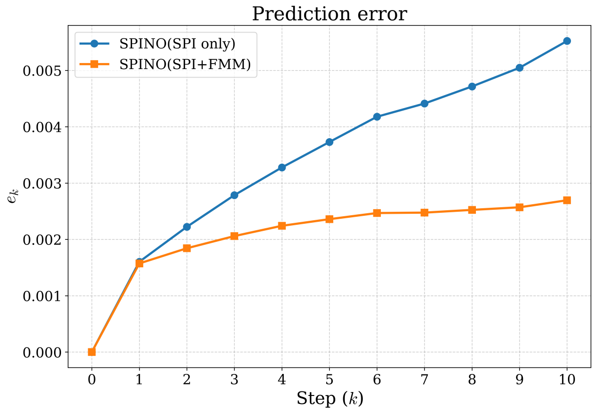

Supplementary Figure 10: Comparison of the step-wise prediction error e_k between the models with and without the Feature Mixture Module (FMM) on the Burgers' equation with varying external forces f.

Feature Interaction Module (FIM). Recall that the recursive error inequality involves both the one-step bias α_k and the amplification factor β_k. The Feature Interaction Module (FIM) is primarily designed to reduce the one-step bias α_k by attenuating the dominance of SPI guidance and allowing network-generated high-level features to jointly determine the dynamic convolution parameters in deeper layers' SPINO block.

Concretely, in the last SPINO block, FIM extends the SPI-based parameterization by incorporating guidance from high-level features generated by the network itself. Instead of letting the dynamic convolution parameters θ depend solely on the SPI representations, FIM introduces an interaction mechanism that blends the SPI with a compact representation of the deep feature $\mathbf{v}_{l-1}$. This yields a refined conditioning for θ in the prediction step:

$$\tilde{x}_{k+1} = \mathcal{G}_\theta(\tilde{x}_k), \qquad \theta \sim p\big(\theta \mid \mathcal{P}(\mathcal{D}(\tilde{x}_k)),\ \mathcal{P}(\mathcal{D}(\tilde{x}_{k-1})),\ \phi(\mathbf{v}_{l-1})\big), \tag{23}$$

where $\phi(\mathbf{v}_{l-1})$ denotes a dimension-aligned summary of the deep feature. In this way, the dominance of SPI guidance is gradually weakened in deeper layers, while the network's own high-level features increasingly participate in determining θ, thereby improving expressivity and reducing the one-step bias α_k.

By allowing $\mathbf{v}_{l-1}$ to co-guide the dynamic convolution, FIM reduces the mismatch between

the effective parameterization and the local state in complex regimes where SPI alone is imperfect, thereby decreasing the one-step bias α_k.

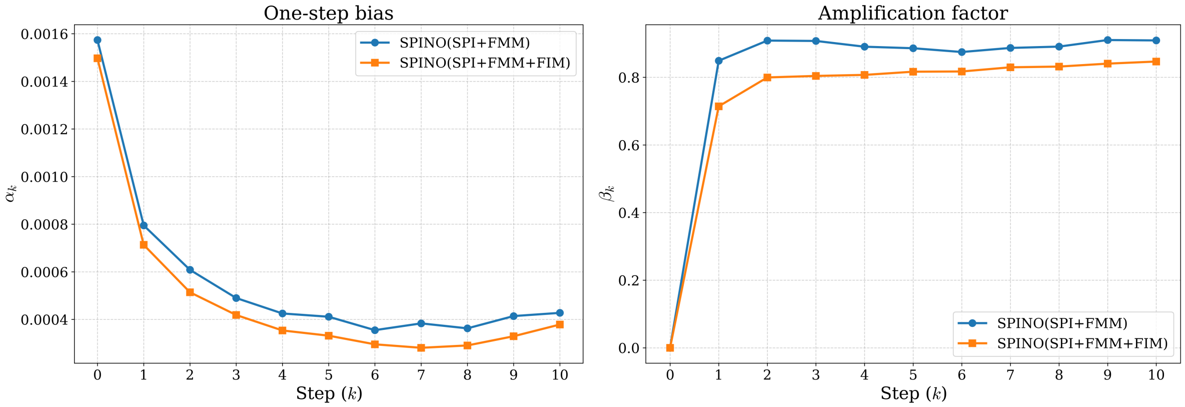

Supplementary Figure 11: Comparison of the one-step error coefficient α_k and amplification factor β_k between the models with and without the Feature Interaction Module (FIM) on the Burgers' equation with varying external forces f.

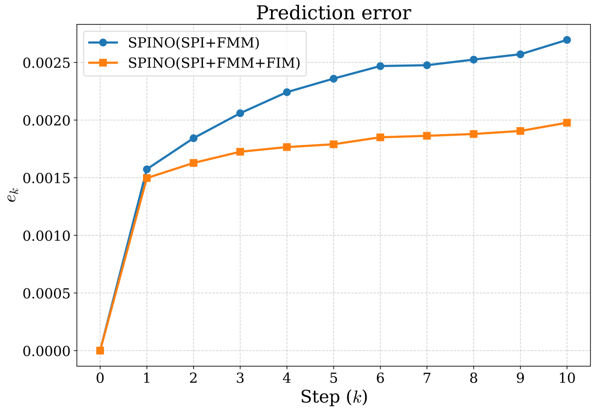

Supplementary Figure 12: Comparison of the step-wise prediction error e_k between the models with and without the Feature Interaction Module (FIM) on the Burgers' equation with varying external forces f.

We also compute α_k and β_k across different steps k, both with and without the module, on the Burgers' equation under varying external forces f. The comparative results are illustrated in Supplementary Figure 11. The results demonstrate that FIM consistently reduces the one-step error coefficient α_k. A decrease in β_k is also observed, which we attribute to the weakened dominance of SPI guidance in deeper layers during auto-regressive prediction. As the influence of noisy or imperfect SPI representations diminishes, the propagation of errors across recursive

steps is effectively alleviated, resulting in a lower amplification factor β_k. The corresponding step-wise prediction error is illustrated in Supplementary Figure 12.

9 Evaluation of SPI-based operator learning in real-world scenario

To demonstrate the applicability of our model in real-world scenario, we conduct experiments on the *ERA5 Sea Surface Temperature (SST)* [10] dataset provided by the European Centre for Medium-Range Weather Forecasts (ECMWF). The dataset contains global sea surface temperature fields at a spatial resolution of $0.25° \times 0.25°$, recorded daily at 00:00 UTC. In this study, we focus on the year 2024, covering the global ocean area between 60°S–60°N and 180°W–180°E. Each grid point represents the mean sea surface temperature at a specific geographical location, and each temporal frame corresponds to one day of observation.

All sequences for training, validation, and testing begin from a fixed initial time of January 1, 2024, with one frame per day. Spatial tiles of size 64×64 are sampled from the SST field using a stride of 16 to ensure regional diversity. The training set contains 1000 samples with 200 consecutive frames each. The validation set, consisting of 200 samples, is a subset of the training data to maintain spatial consistency. The test set covers the same regions but extends to 240 frames, enabling evaluation of temporal extrapolation. This fixed-start and spatially consistent sampling strategy aligns with real-world forecasting practices, where models are trained on historical data and assessed on future predictions within the same domain. The sea surface temperature (SST) data used in this study are essentially gridded and spatially regular. However, to account for irregularities introduced by land–sea boundaries, land grid points are identified and treated separately from ocean regions. During training, the temperature values over land are replaced with the mean sea surface temperature computed from valid ocean areas. This treatment ensures that the model receives spatially continuous input while avoiding the influence of non-physical land values, thereby preserving the overall physical consistency of the dataset.

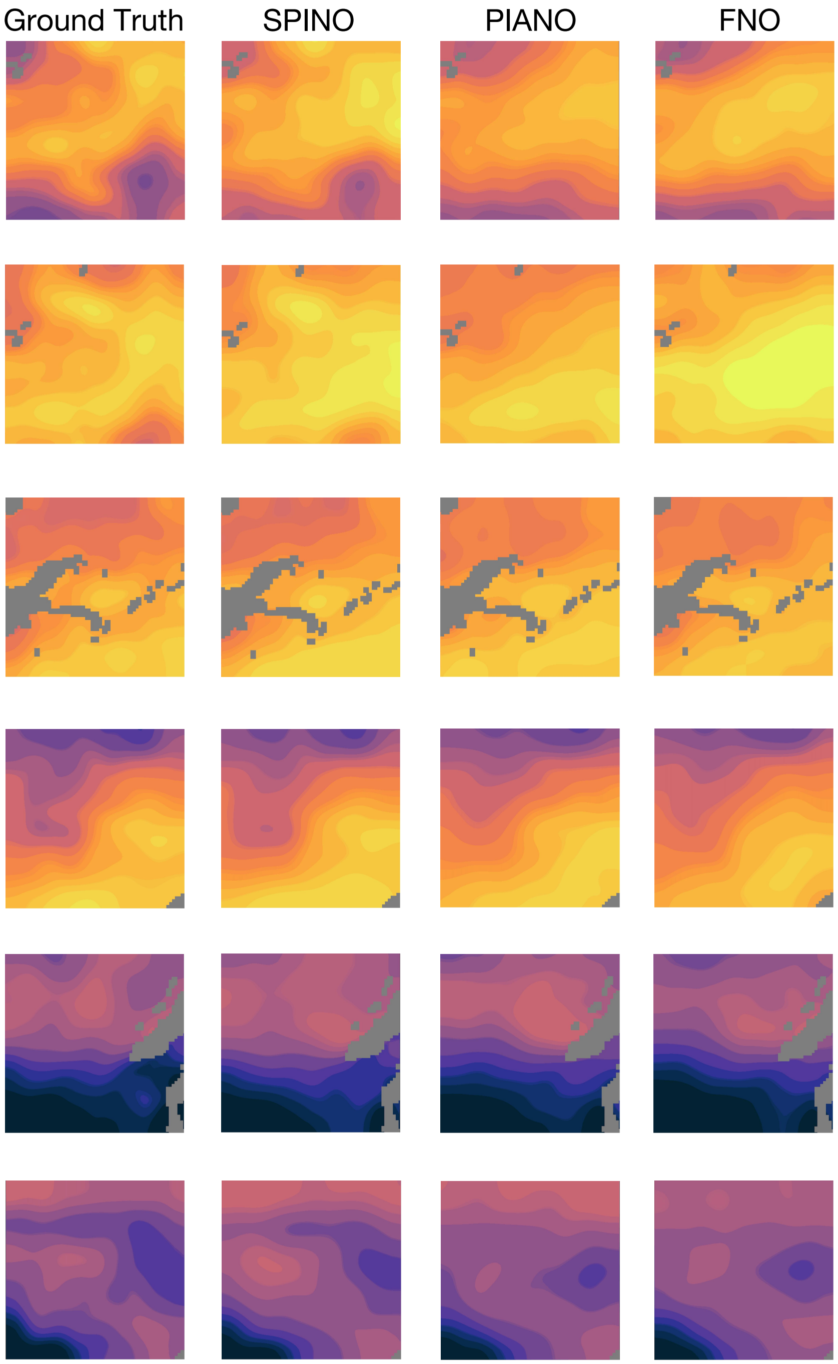

Supplementary Figure 13: Comparison of SST fields from the ground truth and three operator learning models: SPINO, PIANO, and FNO, on January 31. The first column shows the ground-truth SST, while the subsequent columns show the predictions from each model. Gray regions denote land areas, and color indicates the sea surface temperature.

Supplementary Table 10: Comparison of L_2 error (%) for different models on the ERA5 SST dataset in both training and future domains. The best results for each task are highlighted in **bold**.

Method	Physical Invariant	Training domain ($E_{l_2}(\%)$)	Future domain ($E_{l_2}(\%)$)
FNO [2]	-	10.874	22.340
PIANO [8]	static	9.423	19.159
**SPINO (ours)**	sliding	**5.413**	**11.728**

We compare three operator-learning-based models–FNO [2], PIANO [8], and our proposed SPINO–under identical training and testing settings. Quantitative results, summarized in Supplementary Table 10, demonstrate the superior generalization and stability of the proposed SPINO model compared with FNO and PIANO. Complementary qualitative visualizations of sea surface temperature (SST) fields at the same time step, shown in Supplementary Figure 13, further confirm these findings. The results consistently demonstrate that incorporating sliding physical invariant enhances the generalization ability and stability of the proposed SPINO model compared with FNO and PIANO. This improvement may offer practical benefits for real-world ocean forecasting, where dynamic and nonlinear physical processes often influence model stability and prediction accuracy.

10 Other experiments

10.1 1D Burgers' equation with a lower viscosity $\nu = 0.01$

To further evaluate the robustness of the models under more complex dynamics, we conduct additional experiments on the one-dimensional Burgers' equation with a lower viscosity parameter $\nu = 0.01$. This setting produces sharper gradients and more nonlinear behaviors compared with the $\nu = 0.1$ case discussed in the main text. The governing equation is given by:

$$\begin{aligned} \frac{\partial u}{\partial t} &= -u\frac{\partial u}{\partial x} + 0.01\Delta u + 0.1 f(x), \\ x &\in [-\pi, \pi], \quad u(\pm\pi, t) = 0, \end{aligned} \tag{24}$$

where $f(x)$ is chosen from the same set of 14 external forces as in Experiment E1 of the main paper. Except for the viscosity parameter, all configurations follow the 1D setup in the main paper.

Supplementary Table 11 reports the quantitative results of the Burgers' equation with a lower viscosity $\nu = 0.01$. Compared with the $\nu = 0.1$ case presented in the main text, this lower-viscosity setting leads to sharper gradients and thus more challenging prediction tasks, as reflected in the higher error levels across all methods. Among the baseline approaches, FNO shows the largest errors in both training and future domains, while PIANO achieves moderate improvements but still exhibits noticeable error accumulation over time. In contrast, SPINO consistently outperforms both baselines, maintaining the lowest errors and demonstrating superior robustness under the more challenging low-viscosity setting.

Supplementary Table 11: Results of the Burgers' equation with a lower viscosity $\nu = 0.01$. $E_{l_2}(\%)$ and $E_{l_\infty}(\%)$ denote the relative L_2 and L_∞ errors, respectively. The best results for each task are highlighted in **bold**.

Model	Training domain		Future domain	
	$E_{\ell_2}(\%)$	$E_{\ell_\infty}(\%)$	$E_{\ell_2}(\%)$	$E_{\ell_\infty}(\%)$
FNO	$1.044_{\pm\ 0.028}$	$6.244_{\pm\ 0.284}$	$2.919_{\pm\ 0.078}$	$14.612_{\pm\ 0.421}$
PIANO	$0.799_{\pm\ 0.007}$	$4.694_{\pm\ 0.133}$	$2.047_{\pm\ 0.040}$	$12.007_{\pm\ 0.109}$
SPINO(ours)	$\mathbf{0.515_{\pm\ 0.019}}$	$\mathbf{3.814_{\pm\ 0.152}}$	$\mathbf{1.561_{\pm\ 0.060}}$	$\mathbf{10.876_{\pm\ 0.543}}$

To validate the modelling capability of the proposed method on the Burgers' equation with a low viscosity parameter ($\nu = 0.01$), we compare the predicted solution with the ground truth, as visualized in Supplementary Figure 14. Under low-viscosity conditions, the nonlinear convection term dominates, causing the Burgers' equation to develop multiple shock waves and sharp gradients during temporal evolution. Panel (a) shows the spatiotemporal evolution of the ground-truth solution, where the reduced viscosity leads to pronounced gradients and nonlinear interactions. Panel (b) presents the prediction from SPINO, which closely matches the ground truth and demonstrates the model's ability to capture complex dynamics with high fidelity. The absolute error distribution in Panel (c) remains uniformly low, with only slight accumulation near steep-gradient regions, confirming the stability of the predictions.

Finally, panel (d) depicts cross-sectional curves at selected times ($t = 1.0, 2.0, \ldots, 6.0$). Overall, these results confirm that SPINO achieves accurate and stable long-term predictions even in challenging low-viscosity Burgers' equation.

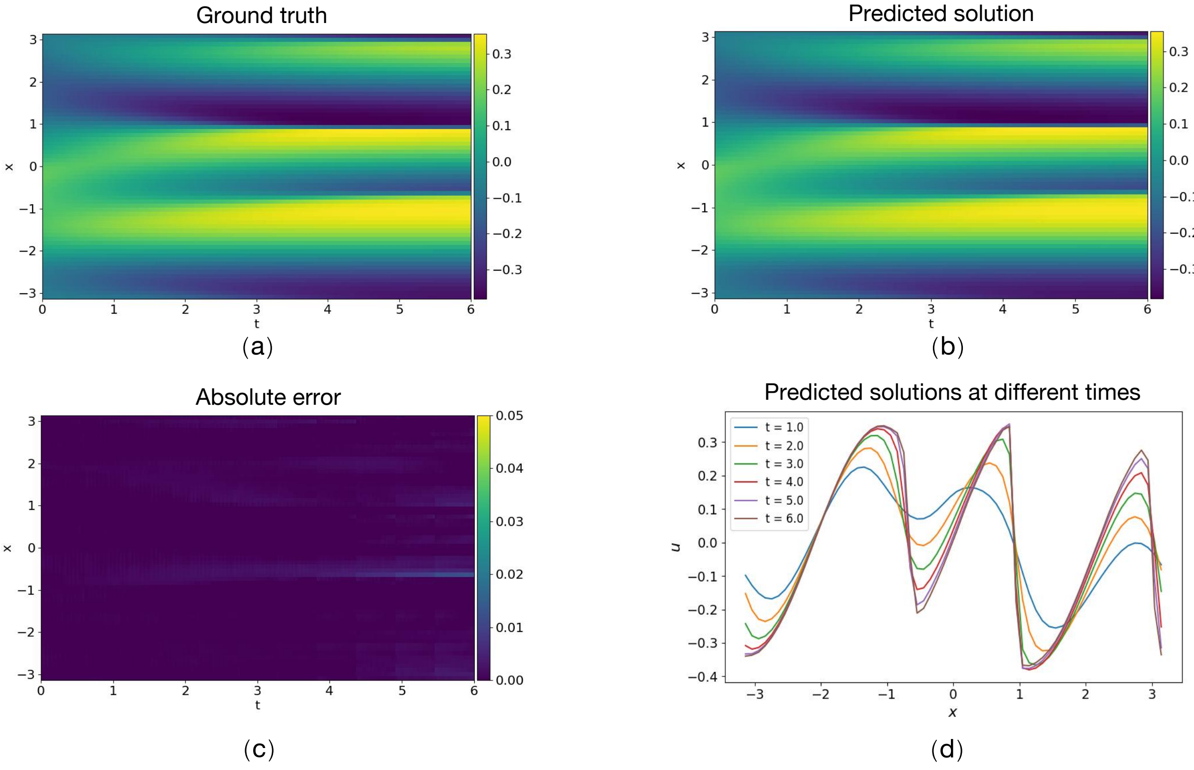

Supplementary Figure 14: Visualization of 1D burgers' equation results.

10.2 1D Burgers' equation with diverse initial conditions

In this experiment, the governing equation, computational domain, boundary conditions, and numerical discretization scheme remain consistent with the E1 dataset described in the main manuscript. The only modification is in the generation of the initial conditions, which are designed to enhance diversity and reduce statistical redundancy. Specifically, the initial field $u(x, 0)$ is generated using a Gaussian random field (GRF) with a Matérn-type power spectral density

$$S(k) \propto \left(4\pi^2 k^2 + \tau^2\right)^{-\alpha/2}, \tag{13}$$

which follows the general form of the Matérn class spectral density [11]. The smoothness parameter α and the length-scale parameter τ are independently drawn from uniform distributions $\alpha \sim \mathcal{U}(1.6,\, 3.2)$ and $\tau \sim \mathcal{U}(1.0,\, 6.0)$, respectively. This randomization ensures that some realizations are smoother while others are more oscillatory, thereby collectively covering a broad spectrum of spatial frequencies. To further enhance the diversity of initial conditions, a correlation gating mechanism is applied: for each candidate field, the absolute Pearson correlation coefficient with all previously accepted samples is computed, and the candidate is resampled if this coefficient exceeds 0.9. This thresholding procedure eliminates both highly correlated and anti-correlated realizations. Although opposite in sign, these realizations typically exhibit comparable spectral characteristics and temporal evolution patterns in PDE-governed systems. Reducing correlations among initial conditions is essential for assessing the true generalization ability of operator learning models, as highly redundant states may lead to overly optimistic performance estimates.

To quantitatively and visually assess dataset diversity, we compute pairwise Pearson cor-

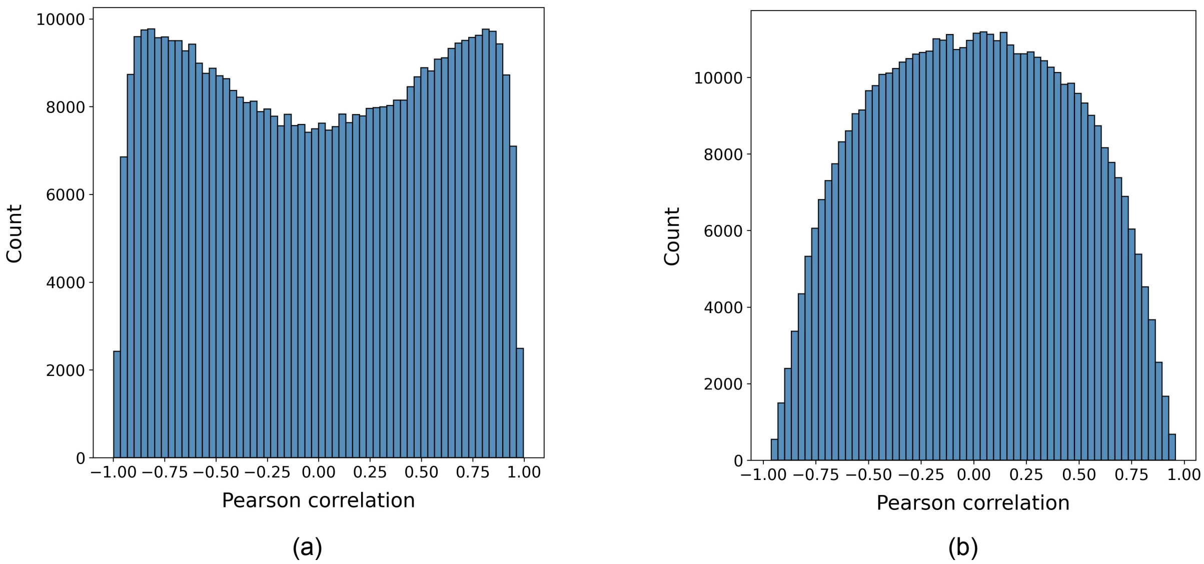

Supplementary Figure 15: Analysis of the diversity of initial conditions in the training dataset. Pearson correlation values range from -1 (strong negative) to 1 (strong positive), with 0 indicating weak or no correlation. (a) Pairwise correlations among the original initial conditions, showing relatively concentrated values. (b) Pairwise correlations after introducing diverse initial conditions, with a broader spread that reflects greater variability and diversity.

Supplementary Table 12: Results of the Burgers' equation with and without diverse initial conditions. $E_{l_2}(\%)$ and $E_{l_\infty}(\%)$ denote the relative L_2 and L_∞ errors, respectively. The best results for each task are highlighted in **bold**.

Data	Training domain		Future domain	
	$E_{\ell_2}(\%)$	$E_{\ell_\infty}(\%)$	$E_{\ell_2}(\%)$	$E_{\ell_\infty}(\%)$
Burgers' equation w/o diverse initial conditions	$\mathbf{0.286}_{\pm\ \mathbf{0.070}}$	$\mathbf{0.452}_{\pm\ \mathbf{0.001}}$	$\mathbf{0.244}_{\pm\ \mathbf{0.005}}$	$\mathbf{0.403}_{\pm\ \mathbf{0.004}}$
Burgers' equation w/ diverse initial conditions	$0.360_{\pm\ 0.024}$	$0.454_{\pm\ 0.019}$	$0.729_{\pm\ 0.021}$	$0.870_{\pm\ 0.015}$

relations among the $N = 1000$ training initial conditions, yielding $N(N-1)/2 = 499{,}500$ unique sample pairs. A visual analysis of the pairwise Pearson correlations among the samples is provided in Supplementary Figure 15. As shown in panel (a), the histogram for the original initialization strategy reveals that many sample pairs exhibit relatively high correlations, indicating limited diversity. In contrast, panel (b) demonstrates that with the modified initialization strategy, the 499,500 pairwise correlations are broadly distributed across $[-1, 1]$, and only a small fraction of pairs attain values close to ± 1. This visual evidence confirms that the modified strategy substantially improves the diversity of the training dataset compared to the original design.

We further evaluated the robustness of the model by training with and without diverse initial conditions. As shown in Supplementary Table 12, training without diverse initial conditions leads to higher accuracy, whereas incorporating diverse initial conditions results in a slight reduction in precision due to the increased variability. Nonetheless, the model consistently demonstrates strong performance and generalization capability.

10.3 1D Diffusion-sorption equation

The Diffusion-sorption equation describes a diffusion process hindered by a sorption mechanism. The equation is expressed as:

$$\partial_t u(t,x) = D/R(u)\partial_{xx}u(t,x), \quad x \in (0,1), t \in (0,500], \tag{25}$$

where D denotes the effective diffusion coefficient, and R is the retardation factor used to characterize the inhibitory effect of sorption on the diffusion process. It is important to note

that R is a function of the variable u. Here, $u(t,x)$ represents the solute concentration (e.g., pollutant concentration) at position x and time t, which is the primary state variable of the system. Its spatial and temporal variations reflect the transport and accumulation of the solute in the porous medium, and its dependency in $R(u)$ introduces nonlinearity into the diffusion-sorption dynamics. This equation is applicable to various real-world scenarios, with one of the most typical examples being the modeling of contaminant transport in groundwater. The equation is influenced by the retardation factor R, which, based on the Freundlich adsorption isotherm, depends on the variable u [12]:

$$R(u) = 1 + \frac{1-\phi}{\phi}\rho_s k n_f u^{n_f-1}, \tag{26}$$

where $\phi = 0.29$ denotes the porosity of the porous medium, $\rho_s = 2880$ is the bulk density, $k = 3.5 \times 10^{-4}$ is the Freundlich adsorption coefficient, $n_f = 0.874$ is the Freundlich exponent, and the effective diffusion coefficient is given by $D = 5 \times 10^{-4}$. The initial conditions are generated using a uniform distribution, i.e., $u(0,x) \sim \mathcal{U}(0, 0.2)$, where $x \in (0,1)$. The experimental data provided are discretized in space and time as $N_x = 64$ and $N_t = 501$, respectively, and a downsampled version in the time dimension ($N_t = 101$) is provided for model training.

This example is noteworthy for several reasons. First, the diffusion coefficient exhibits nonlinearity due to its dependence on u, and Equation (26) reveals a singularity when $u = 0$. Second, the problem is highly relevant to real-world applications, particularly groundwater contaminant transport, where the use of machine learning methods is still uncommon. Third, instead of the commonly used zero or periodic boundary conditions that are easily managed in convolutional models, we impose more complex conditions: specifically, $u(t,0) = 1.0$ and $u(t,1) = D\partial_x u(t,1)$. The latter is especially challenging because it involves a derivative rather than a fixed value. To generate the dataset, we solve the problem with a standard finite volume method.

To validate the modelling capability of the proposed method in Diffusion-sorption problems, a comprehensive comparison is made between the predicted solution and ground truth, and the relevant results are visualised as shown in Supplementary Figure 16. Specifically, panel (a)

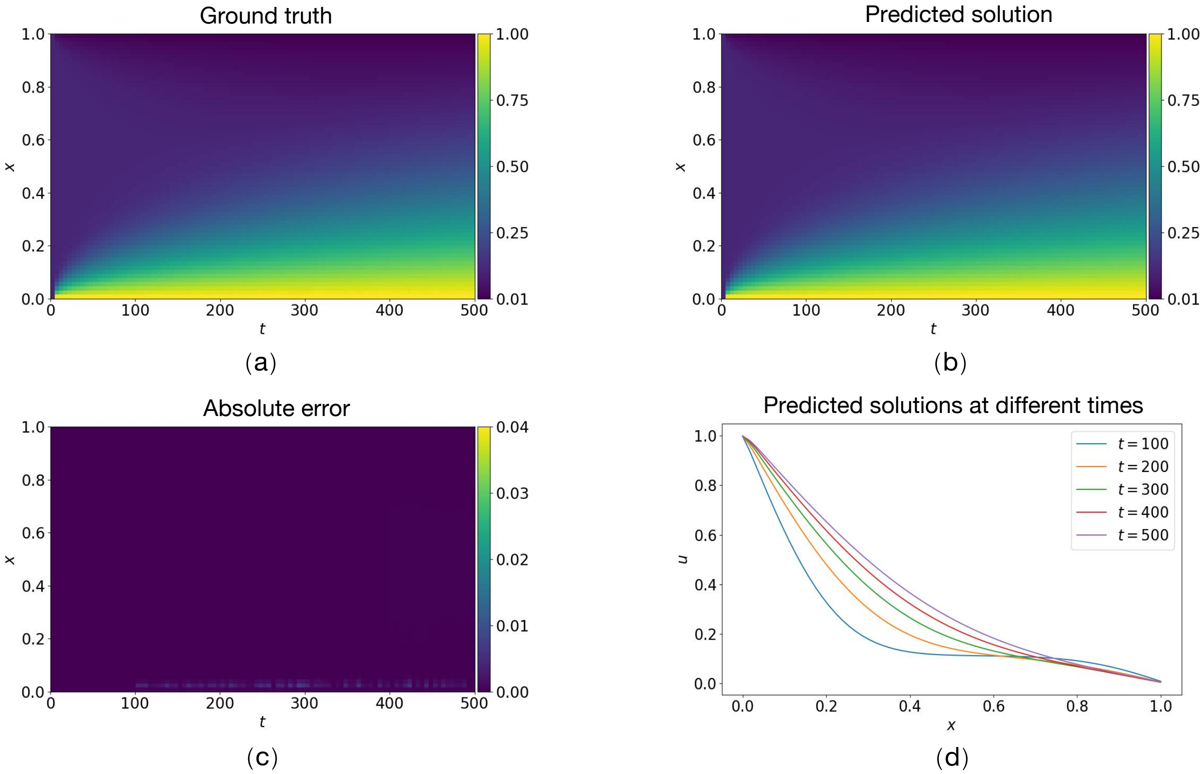

Supplementary Figure 16: Visualization of 1D Diffusion-sorption equation results.

illustrates the spatiotemporal evolution of the ground truth, revealing a progressively expanding and attenuating diffusion pattern over time. Panel (b) shows the predicted solution provided by the model under the same conditions, whose overall distribution is highly consistent with the exact solution, indicating that the model can accurately capture the spatial distribution characteristics of the solution as it evolves over time. Furthermore, panel (c) shows the absolute error distribution between the predicted solution and the true solution. The overall error is low, with only slight accumulation near the boundary and in the later time period, verifying the stability and reliability of the model's predictions. Panel (d) presents the predicted cross-sectional curves at five time points ($t = 100, 200, 300, 400, 500$), showing that the amplitude of the solution decreases over time with smooth spatial variations, indicating that the model can accurately fit the dynamic process. Overall, the model demonstrates strong generalization and high accuracy in spatiotemporal diffusion systems.

10.4 2D Wave equation

The classical 2D Wave equation models the propagation of waves in a homogeneous medium (e.g. water surface) and can be written as

$$\frac{\partial^2 u}{\partial t^2} = \omega^2 \nabla^2 u, \tag{27}$$

where u describes the distance from equilibrium and ω represents the material-dependent speed of propagation. It is a linear, second-order PDE that has been widely used in scientific machine learning [13, 14].

The spatial domain of the simulation is set to $\Omega = [0, 1] \times [0, 1]$ and the temporal domain to $T = [1, 200]$. We initialize the state of the Wave equation using zeros for the velocity field $\frac{\partial u}{\partial t}$ and a sum of Gaussian curves for the displacement field u, similar to what has been used in related work [14, 15]. The Gaussian initialization is constructed as

$$I(x, y) = \sum_{i=1}^{K} A_i \exp\left(-\frac{(x - \mu_{ix})^2 + (y - \mu_{iy})^2}{\sigma^2}\right), \tag{28}$$

where the positions (μ_{ix}, μ_{iy}) of each Gaussian are uniformly sampled from the domain Ω, while amplitudes A_i are drawn uniformly from $[-1, 1]$. We fix $K = 5$ and $\sigma = 0.15$ respectively. For numerical discretization, the spatial domain Ω is represented on a 64×64 grid, giving a cell size of $\Delta x = \Delta y = 0.0156$. Temporal integration is performed using the method of lines [15], with the Explicit Runge–Kutta method of order 5(4) [16] as the ODE solver. This setup provides a high-fidelity reference solution for evaluating the predictive accuracy of operator learning methods.

Supplementary Figure 17 presents a comparison of the ground truth solution, the SPINO predictions, and the corresponding absolute errors across representative time steps. Within the training domain ($T \leq 160$), SPINO accurately captures the evolution of the displacement field, with errors remaining uniformly low across the domain. In the future domain ($T > 160$), SPINO continues to produce stable predictions that preserve the dominant wave structures, with only minor local deviations. The error plots confirm that these discrepancies remain bounded

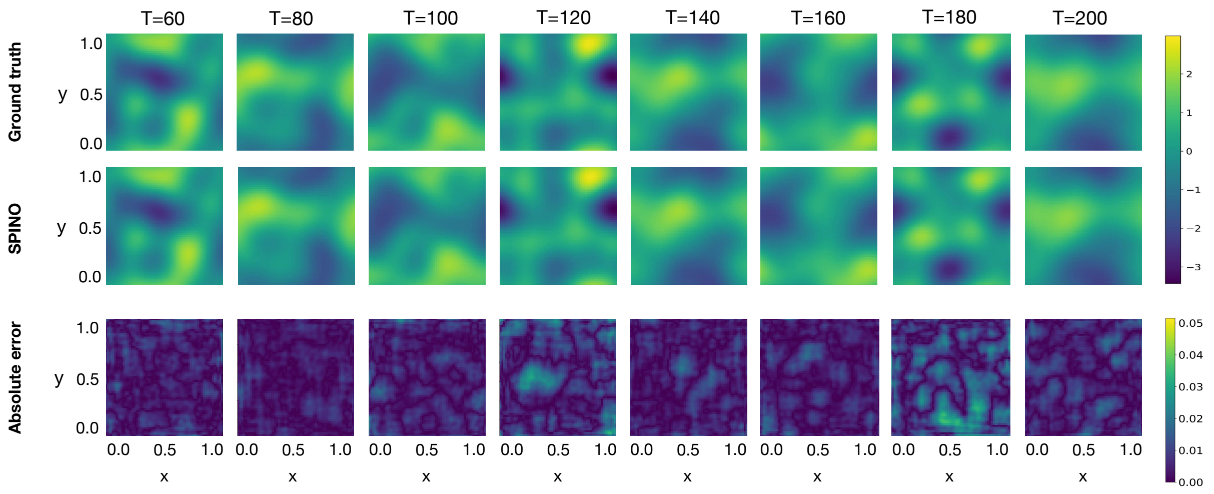

Supplementary Figure 17: Comparison of the displacement fields in the 2D Wave equation from $T = 1$ to $T = 200$ on the periodic domain $\Omega = [0, 1]^2$. Note that early times ($T \leq 160$) correspond to the training domain, while later times ($T > 160$) represent the future domain.

and do not accumulate destructively over long horizons. Overall, these results highlight that SPINO effectively models oscillatory wave dynamics, maintaining both stability and accuracy in forecasting.

10.5 2D Shallow water equation

The Shallow water equation, derived from the compressible Navier–Stokes equation, offers a suitable framework for modelling free surface flow problems. In the PDEBENCH dataset [17], unlike the commonly adopted periodic boundary conditions in FNO benchmarks, the Shallow water equation is implemented with Neumann boundary conditions, which make the problem more realistic and challenging. In two dimensions, it takes the form of the following system of hyperbolic partial differential equations:

$$\begin{aligned} \partial_t h + \partial_x hu + \partial_y hv &= 0, \\ \partial_t hu + \partial_x \left(u^2 h + \tfrac{1}{2} g_r h^2\right) &= -g_r h \partial_x b, \\ \partial_t hv + \partial_y \left(v^2 h + \tfrac{1}{2} g_r h^2\right) &= -g_r h \partial_y b, \end{aligned} \tag{29}$$

where u and v represent the horizontal and vertical velocities, respectively, h describes the water depth, and b describes the spatial variation in water depth topography. hu and hv

can be interpreted as directional momentum components, and g_r describes the gravitational acceleration. Application examples include simulations of tsunamis or general flood events. The shallow water equations are simulated over the temporal domain $T = [0, 1]$, with a resolution of 100 time steps.

The shallow water equation benchmark in this study features a radial dam break scenario. In a square domain $\Omega = [-2.5, 2.5]^2$, the water depth is initialized as a circular protrusion at the center of the domain:

$$h(t = 0, x, y) = \begin{cases} 2.0, & \text{for } r < \sqrt{x^2 + y^2} \\ 1.0, & \text{for } r \geq \sqrt{x^2 + y^2} \end{cases}, \tag{30}$$

where the radius r is randomly sampled from $\mathcal{U}(0.3, 0.7)$. To generate the dataset, the PyClaw [18] Python package is used to simulate this problem, which provides a comprehensive finite volume solver.

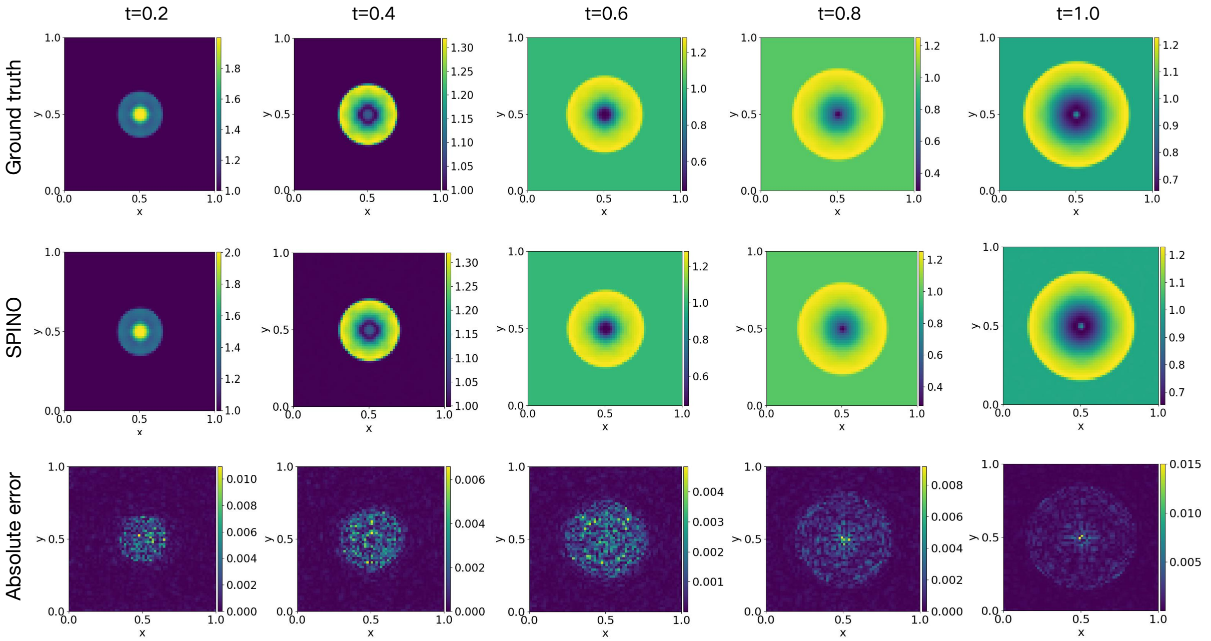

Supplementary Figure 18: Predicted height field results of the 2D Shallow water equation.

In the radial dam break problem of the Shallow water equation, SPINO demonstrates robust spatio-temporal generalization capabilities. As shown in Supplementary Figure 18, the

evolution of the height field at five time points $t = \{0.2, 0.4, 0.6, 0.8, 1.0\}$ is visualized to compare SPINO's predictions with the ground truth. The results indicate that SPINO accurately captures the radial propagation of the disturbance from the center, with the predicted solution closely matching the true solution, particularly at the early stages ($t = 0.2$ and $t = 0.4$).

As time progresses to $t = 0.6$ and beyond, although the range of the disturbance wavefront gradually expands, the model can still accurately depict the spatial distribution of height. However, the prediction error slightly increases in the edge regions of the disturbance, indicating that SPINO experiences some local accuracy loss when handling shock propagation or regions of rapid change. Overall, SPINO maintains high prediction accuracy in this Shallow water equation problem with highly nonlinear characteristics, validating its effectiveness and robustness in complex dynamic system modelling tasks.

11 Computational efficiency analysis

This section provides a detailed analysis of the computational efficiency of the proposed model in comparison to baseline methods, addressing both training time differences and inference performance.

11.1 Training time considerations

The performance metrics and training time results for PIANO and other baseline models presented in the main text are directly adopted from their original publications to ensure fairness and reproducibility. It should be noted that these results were obtained under different hardware configurations. Specifically, the PIANO baseline was trained on an NVIDIA A100 GPU, while our model was trained on an NVIDIA V100 GPU. The hardware difference have contributed to the longer training time observed for our model.

11.2 Inference efficiency analysis

To ensure an equitable comparison across all methods, the PIANO model was reproduced on the same NVIDIA V100 GPU platform used for our experiments. Supplementary Table 13 provides

a comprehensive comparison including predictive accuracy, model parameters, training time per epoch, and inference time measured under different conditions.

Supplementary Table 13: Comparison of model performance and computational efficiency on the datasets E1-E5. The descriptions of E1-E5 can be found in the main text.

**Dataset**	**Model**	**Error (E_{l_2}(%))**		**Training Time (s)**	**Inference Time (s)**		**Parameters (M)**
		**Training domain**	**Future domain**	**(per epoch)**	**All-samples**	**Per-sample**	
E1	PIANO+FNO	$0.471_{\pm 0.021}$	$0.604_{\pm 0.018}$	0.629	0.063	0.034	0.762
	Full SPINO	$0.286_{\pm 0.070}$	$0.325_{\pm 0.073}$	0.706	0.075	0.046	1.118
E2	PIANO+FNO	$4.820_{\pm 0.083}$	$8.949_{\pm 0.298}$	0.311	0.071	0.039	0.762
	Full SPINO	$2.996_{\pm 0.113}$	$6.682_{\pm 0.223}$	0.329	0.089	0.058	1.118
E3	PIANO+FNO	$0.349_{\pm 0.019}$	$0.511_{\pm 0.018}$	0.457	0.069	0.033	0.763
	Full SPINO	$0.147_{\pm 0.017}$	$0.377_{\pm 0.043}$	0.575	0.080	0.062	1.118
E4	PIANO+FNO	$4.672_{\pm 0.362}$	$17.341_{\pm 0.538}$	0.536	0.932	0.763	2.020
	Full SPINO	$2.904_{\pm 0.002}$	$16.048_{\pm 0.073}$	2.471	2.048	0.795	11.873
E5	PIANO+FNO	$1.489_{\pm 0.937}$	$1.600_{\pm 0.858}$	0.945	0.967	0.703	2.020
	Full SPINO	$0.385_{\pm 0.022}$	$0.665_{\pm 0.037}$	2.509	2.044	0.774	11.873

For the 1D problems (E1–E3), the training and inference times of the proposed model remain comparable to those of PIANO+FNO, indicating that the additional architectural components introduce minimal computational overhead in low-dimensional settings. In contrast, for the more computationally demanding 2D problems, the impact of the added modules becomes more pronounced during training. Taking E4 as an example, the proposed model increases the training time per epoch (2.471 s vs. 0.536 s for PIANO+FNO). Nevertheless, the inference efficiency, a more critical indicator for practical applications, remains highly competitive. The inference time comparison reveals two important aspects:

- **All-samples inference**: When processing the entire test set as a single batch, the time difference becomes more noticeable due to hardware memory limitations, though this scenario is less representative of real-world usage.

- **Per-sample inference**: Under realistic deployment conditions where inference is typically performed on individual samples, both methods achieve comparable efficiency.

These results validate the practical feasibility of the proposed approach, demonstrating that the enhanced modeling capabilities do not compromise deployment efficiency in realistic application scenarios.

References

[1] Y. Chen, X. Dai, M. Liu, D. Chen, L. Yuan, and Z. Liu, "Dynamic convolution: Attention over convolution kernels," in *Proceedings of the IEEE/CVF conference on computer vision and pattern recognition*, pp. 11030–11039, 2020.

[2] Z. Li, N. Kovachki, K. Azizzadenesheli, B. Liu, K. Bhattacharya, A. Stuart, and A. Anandkumar, "Fourier neural operator for parametric partial differential equations," in *International Conference on Learning Representations*, 2021.

[3] K. Cho, B. Van Merriënboer, C. Gulcehre, D. Bahdanau, F. Bougares, H. Schwenk, and Y. Bengio, "Learning phrase representations using rnn encoder-decoder for statistical machine translation," in *Conference on Empirical Methods in Natural Language Processing (EMNLP 2014)*, 2014.

[4] Z. Liu, Y. Lin, Y. Cao, H. Hu, Y. Wei, Z. Zhang, S. Lin, and B. Guo, "Swin transformer: Hierarchical vision transformer using shifted windows," in *Proceedings of the IEEE/CVF international conference on computer vision*, pp. 10012–10022, 2021.

[5] D. Soydaner, "Attention mechanism in neural networks: where it comes and where it goes," *Neural Computing and Applications*, vol. 34, no. 16, pp. 13371–13385, 2022.

[6] S. Imambi, K. B. Prakash, and G. Kanagachidambaresan, "Pytorch," *Programming with TensorFlow: solution for edge computing applications*, pp. 87–104, 2021.

[7] J. Brandstetter, D. Worrall, and M. Welling, "Message passing neural pde solvers," *arXiv preprint arXiv:2202.03376*, 2022.

[8] R. Zhang, Q. Meng, and Z.-M. Ma, "Deciphering and integrating invariants for neural operator learning with various physical mechanisms," *National Science Review*, vol. 11, no. 4, p. nwad336, 2024.

[9] T. Chen, S. Kornblith, M. Norouzi, and G. Hinton, "A simple framework for contrastive learning of visual representations," in *International conference on machine learning*, pp. 1597–1607, PmLR, 2020.

[10] H. Hersbach, B. Bell, P. Berrisford, G. Biavati, A. Horányi, J. Muñoz Sabater, J. Nicolas, C. Peubey, R. Radu, I. Rozum, and et al., "Era5 hourly data on single levels from 1979 to present." Copernicus Climate Change Service (C3S) Climate Data Store (CDS), 2018.

[11] C. E. Rasmussen and C. K. I. Williams, *Gaussian Processes for Machine Learning*. Cambridge, MA: MIT Press, 2006.

[12] G. Limousin, J.-P. Gaudet, L. Charlet, S. Szenknect, V. Barthès, and M. Krimissa, "Sorption isotherms: a review on physical bases, modeling and measurement," *Applied Geochemistry*, vol. 22, pp. 249–275, 2007.

[13] M. Karlbauer, S. Otte, H. P. Lensch, T. Scholten, V. Wulfmeyer, and M. V. Butz, "Inferring, predicting, and denoising causal wave dynamics," in *International Conference on Artificial Neural Networks*, pp. 566–577, Springer, 2020.

[14] A. Dulny, A. Hotho, and A. Krause, "Dynabench: A benchmark dataset for learning dynamical systems from low-resolution data," in *Joint European Conference on Machine Learning and Knowledge Discovery in Databases*, pp. 438–455, Springer, 2023.

[15] A. Dulny, A. Hotho, and A. Krause, "Neuralpde: modelling dynamical systems from data," in *German Conference on Artificial Intelligence (Künstliche Intelligenz)*, pp. 75–89, Springer, 2022.

[16] J. R. Dormand and P. J. Prince, "A family of embedded runge-kutta formulae," *Journal of computational and applied mathematics*, vol. 6, no. 1, pp. 19–26, 1980.

[17] M. Takamoto, T. Praditia, R. Leiteritz, D. MacKinlay, F. Alesiani, D. Pflüger, and M. Niepert, "Pdebench: An extensive benchmark for scientific machine learning," *Advances in Neural Information Processing Systems*, vol. 35, pp. 1596–1611, 2022.

[18] D. I. Ketcheson, K. Mandli, A. J. Ahmadia, A. Alghamdi, M. Q. De Luna, M. Parsani, M. G. Knepley, and M. Emmett, “Pyclaw: accessible, extensible, scalable tools for wave propagation problems,” *SIAM Journal on Scientific Computing*, vol. 34, pp. C210–C231, 2012.
